# Large Language Models for Health Care Text Classification: Systematic Review

**DOI:** 10.2196/79202

**Published:** 2026-02-11

**Authors:** Hajar Sakai, Sarah S Lam

**Affiliations:** 1 Binghamton University Binghamton, NY United States

**Keywords:** large language models, text classification, health care, natural language processing, neural networks, systematic review

## Abstract

**Background:**

Large language models (LLMs) have fundamentally transformed approaches to natural language processing tasks across diverse domains. In health care, accurate and cost-efficient text classification is crucial—whether for clinical note analysis, diagnosis coding, or other related tasks—and LLMs present promising potential. Text classification has long faced multiple challenges, including the need for manual annotation during training, the handling of imbalanced data, and the development of scalable approaches. In health care, additional challenges arise, particularly the critical need to preserve patient data privacy and the complexity of medical terminology. Numerous studies have leveraged LLMs for automated health care text classification and compared their performance with traditional machine learning–based methods, which typically require embedding, annotation, and training. However, existing systematic reviews of LLMs either do not specialize in text classification or do not focus specifically on the health care domain.

**Objective:**

This research synthesizes and critically evaluates the current evidence in the literature on the use of LLMs for text classification in health care settings.

**Methods:**

Major databases (eg, Google Scholar, Scopus, PubMed, ScienceDirect) and other resources were queried for papers published between 2018 and 2024, following the PRISMA (Preferred Reporting Items for Systematic Reviews and Meta-Analyses) guidelines, resulting in 65 eligible research articles. These studies were categorized by text classification type (eg, binary classification, multilabel classification), application (eg, clinical decision support, public health and opinion analysis), methodology, type of health care text, and the metrics used for evaluation and validation.

**Results:**

The systematic review includes 65 research articles published between 2020 and Q3 2024, showing a significant increase in publications over time, with 28 papers published in Q1-Q3 2024 alone. Fine-tuning was the most common LLM-based approach (35 papers), followed by prompt engineering (17 papers). BERT (Bidirectional Encoder Representations from Transformers) variants were predominantly used for multilabel classification (50%), whereas closed-source LLMs were most commonly applied to binary (44.0%) and multiclass (30.6%) classification tasks. Clinical decision support was the most frequent application (29 papers). Over 80% of studies used English-language datasets, with clinical notes being the most common text type. All studies employed accuracy-related metrics for evaluation, and the findings consistently showed that LLMs outperformed traditional machine learning approaches in health care text classification tasks.

**Conclusions:**

This review identifies existing gaps in the literature and highlights future research directions for further investigation.

## Introduction

### Background

Large language models (LLMs) are currently a major technology trend that is accessible to nearly everyone. Their landscape is expanding rapidly, with new models being introduced at an accelerated pace. These models vary across open- and closed-source paradigms, as well as general-purpose and domain-specific applications, with some designed to be multilingual, multimodal, or both. Certain foundation LLMs are also fine-tuned to address specific tasks while targeting selected domains. Organizations, along with individuals, continually explore how to efficiently leverage these LLMs to extract insights and relevant information that could advance decision-making processes from continuously accumulating textual data; the health care industry is no exception. In this section, language modeling is reviewed, the importance of text classification is discussed, and the research objectives and structure are outlined.

### Language Modeling

**Figure 1:**
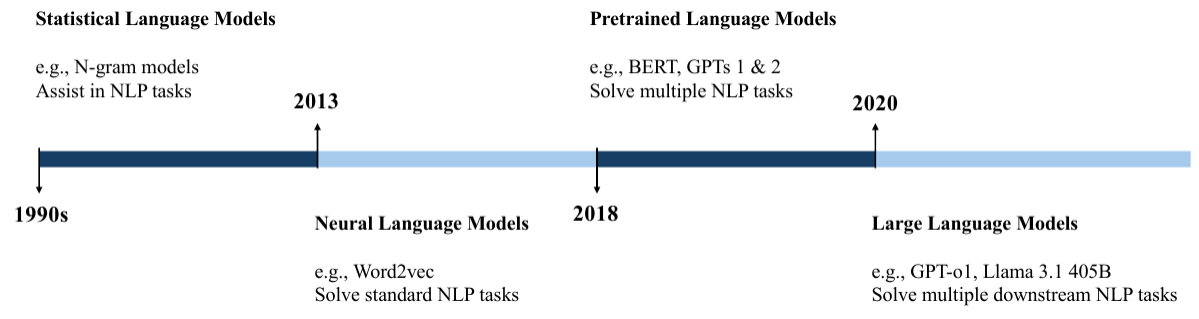
The evolution of language modeling. BERT: Bidirectional Encoder Representations from Transformers; NLP: natural language processing.

Language modeling involves building a mathematical model with statistical probabilities that represent the structure and sequence of tokens or words. The currently most widely used language models (LMs) are autoregressive, in which the distribution over tokens is decomposed into conditional probabilities; in other words, these models can predict the next token given the previously provided context and generated tokens. LMs are characterized by both syntactic and semantic knowledge. Research on language modeling has evolved through 4 main generations (ie, Figure 1), each characterized by a specific task-solving capacity, with the usefulness of the developed LMs increasing over time [1].

In the 1990s, statistical language models (SLMs) were introduced. These models rely on the Markov assumption, which states that the probability of a word depends only on the previous words representing the context. The frequency and co-occurrence of words in a large corpus are used to calculate the probability of a word sequence. One well-known instance of these models is N-gram LMs, in which the context length is fixed. However, these models face challenges related to data sparsity, particularly in the case of rare word sequences. Additionally, they are unable to capture long-term dependencies because the context length is fixed; moreover, when this length is large, probability estimation accuracy degrades due to the significantly large number of transition probabilities that must be calculated. As a result, SLM applications are limited to assisting and improving certain natural language processing (NLP) tasks.

Around 2013, neural language models (NLMs) were introduced. These models use neural networks to calculate word sequence probabilities and aim to handle natural language by learning word representations as continuous vectors. The learned representations capture latent patterns and semantics, as well as long-term dependencies, thereby overcoming the challenges faced by SLMs. Word2Vec, introduced in 2013, is one of the most popular NLMs and is commonly used to learn features from text. Their applications include solving a range of standard NLP tasks. However, because these models are based on deep learning, the interpretability and explainability of the learned representation vectors remain challenging. In addition, these models are computationally intensive and require large corpora for training. It is also worth noting that these learned representations are static.

At the beginning of 2018, pretrained language models (PLMs) were introduced. Given that NLMs provide static word representations, a shift toward learning context-aware word representations using PLMs was observed. This was achieved by pretraining models (eg, bidirectional long short-term memory in the case of Embeddings from Language Models) to generate task-agnostic representations, followed by task-specific fine-tuning. As a result, a new learning paradigm was developed: “pretraining and fine-tuning.” BERT (Bidirectional Encoder Representations from Transformers) and GPT-1 and GPT-2 are regarded as part of the PLM family. As PLM pretraining requires greater computational resources and larger data scales, its limitations include the significant demand for computational resources for both pretraining and fine-tuning. Additionally, these representations are highly dependent on the corpora used for training; therefore, the collected data may introduce bias into the model’s outputs. These models, among others, have demonstrated strong performance when applied to multiple NLP tasks.

In 2020, research on scaling previously introduced PLMs—at both the model and data size levels—peaked with the release of LLMs such as GPT-3 [2] and LaMDA (Language Model for Dialogue Applications) [3], followed by PaLM (Pathways Language Model) [4] and LLaMA (Large Language Model Meta AI) [5]. LLMs stand out from other LMs due to 4 key attributes. First, LLMs are trained on significantly large corpora. Second, LLM architectures are substantially larger than those of PLMs, increasing the number of parameters, which are now typically counted in billions. Third, LLMs support prompt-based completion, making them more accessible and intuitive, particularly through applications such as ChatGPT, announced by OpenAI in 2022, which enables conversational interaction in natural language [6]. Fourth, LLMs not only assist in solving specific tasks, as SLMs do, but can also address multiple real-world tasks, such as clinical notes classification. As a result, the latest generation of LLMs is now considered “general-purpose task solvers” and can be applied to multiple downstream traditional NLP tasks, including text classification.

### Large Language Models

**Figure 2:**
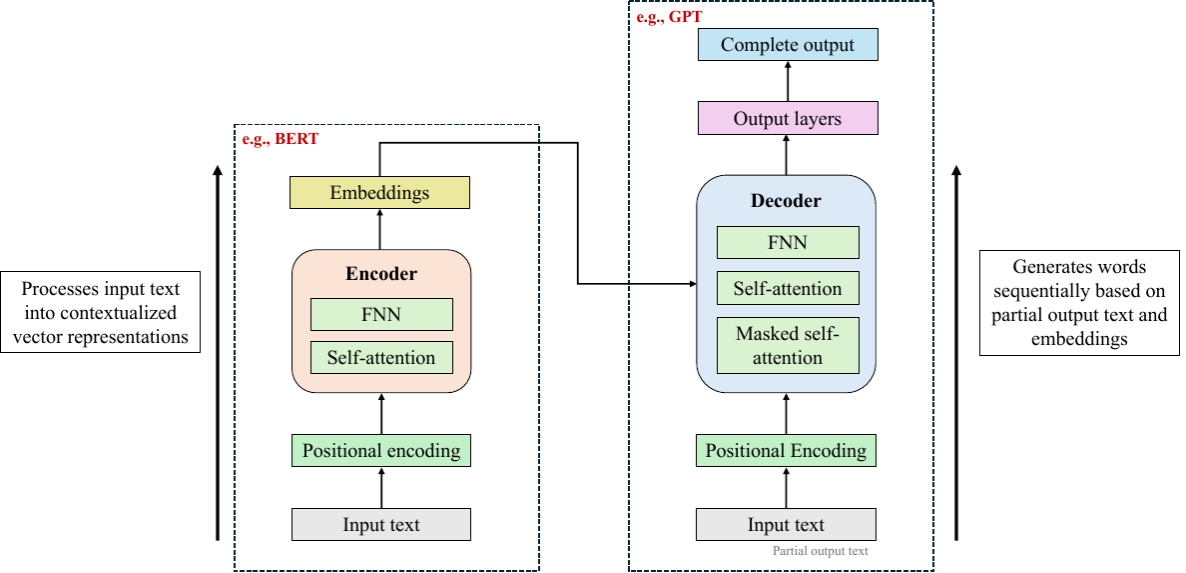
High-level transformer architecture with BERT and GPT as examples. BERT: Bidirectional Encoder Representations from Transformers; FNN: feedforward neural network.

LLMs are deep neural networks, and most modern models are transformer-based. They are trained on large amounts of text data and demonstrate an impressive ability to interact with and generate human-like natural language. These LMs are large in terms of architectural complexity, number of parameters, and the scale of pretraining data. The advent of the transformer underpins the revolution currently witnessed in NLP, in which the attention mechanism [7] incorporated into its architecture enables LLMs to focus on different parts of the text input when generating each part of the output. This is achieved by weighting the relative importance of each token in a text sequence. The transformer architecture was originally developed for machine translation and comprises 2 components: an encoder and a decoder, as shown in Figure 2. Both components consist of multihead self-attention and feedforward neural network layers. Two key LMs, BERT and GPT, are also based on variants of the transformer architecture: the former is built on the encoder component and trained for masked word prediction, whereas the latter is based on the decoder component and trained for text generation, 1 word at a time [8].

Developing an LLM from scratch is highly resource-intensive, requiring immense computational power, large datasets (for which annotation is often needed during fine-tuning), and substantial financial investment. These resources are necessary for data sampling, pretraining the foundation model, and fine-tuning it for specific tasks such as text classification [8]. Consequently, numerous studies have chosen to leverage existing LLMs which, although not originally designed for text classification, have demonstrated the ability to perform this task effectively—particularly in health care settings, where the resources needed to develop a tailored foundation model are usually limited. It is worth noting that, in this systematic review, PLMs such as BERT and its variants are referred to as LLMs.

### Text Classification

#### Motivation

The expansion of the internet and the complete digitalization of various domains, such as health care, have triggered the continuous generation of textual data; as a result, vast amounts of text are being accumulated. This situation presents challenges in text data management and analysis, as well as valuable research opportunities. The need to effectively organize textual data and transform them into structured formats has guided research toward exploring the automatic assignment of predefined categories to text, thereby improving access to information.

There are various motivations behind research focused on efficiently categorizing collected text data, particularly because text classification constitutes a foundational task for multiple NLP applications. Sebastiani [9] highlighted its applications as including text indexing for Boolean information retrieval, document categorization and filtering, and word sense disambiguation. In an information retrieval system, text indexing is based on a controllable dictionary in which documents are paired with 1 or more keywords. When the ensemble of vocabulary contained in the controllable dictionary is considered as categories, text indexing—which facilitates information retrieval—can be viewed as a form of text classification. Additionally, this process results in the automated categorization of documents, which can be extended to web pages; using the assigned labels, text filtering is then enabled. In addition, word sense disambiguation is another application of text classification, in which the categories correspond to the senses of an ambiguous word given the context in which it appears. This is particularly useful for other NLP tasks, such as machine translation. Furthermore, text classification is valuable for understanding and enhancing customer or user experience through sentiment analysis [10]. Sentiment analysis is a special case of text classification in which the considered categories are sentiments (eg, positive, negative, mixed, neutral). This highlights the practical applications of text classification, including customer feedback analysis and interpretation. Moreover, spam detection is another motivation for developing effective text classification techniques for email classification [11]. In the same context, content moderation also relies on text classification [12], providing an additional driver for this line of research.

In health care, text classification is gaining importance, particularly as medical textual data are growing at an exponential rate. Text classification enables the automatic extraction of valuable insights from various types of continuously generated health care narratives, such as clinical notes, patients’ feedback, and medical research papers. This is especially crucial because it can improve patient care by leveraging available data that are usually challenging to mine, resulting in more efficient health care systems [13]. There are diverse health care NLP tasks to which text classification can significantly contribute by providing clinical decision support, such as automated diagnosis coding [14] and the identification of patients at risk for certain diseases [15]. These text classification applications are valuable for early intervention and anticipatory treatment planning. With the evolution of machine learning techniques—particularly deep learning methods—applied to text classification, there is promising potential to achieve accuracy rates comparable to human expert annotations without constant human intervention.

#### Text Classification Over Time

Over the decades, the literature has witnessed several stages in the methodologies used for text classification. It began with the development of rule-based approaches [16], in which dictionaries and rules were manually crafted and therefore lacked scalability. This was followed by statistical and machine learning techniques, such as the multinomial naïve Bayes classifier [17], support vector machines [18], decision tree algorithms [19], and ensemble methods such as random forests [20]. It is important to highlight that the application of machine learning models to text classification would not have been possible without the parallel development of representation learning and feature engineering techniques. These methods have, in turn, evolved from simple Bag-of-Words techniques and their extension, N-grams, to term frequency–inverse document frequency, and later to embedding learning methods that capture semantic and syntactic relationships between words, such as Word2Vec, or capture analogies and linear relationships between words, such as GloVe [21]. These developments enabled the vectorization of text data into dense representations and, consequently, unlocked the potential for the successful application of neural networks and deep learning models to text classification. For instance, Socher et al [22] employed a feed-forward network with a recursive autoencoder for binary polarity classification. A few years later, Zhang and LeCun [23] demonstrated that convolutional neural networks can also be effectively used for text classification. As textual data are sequential, recurrent neural networks were subsequently applied to text classification tasks [24]. Attention mechanisms were then introduced, enabling models to identify the most relevant parts of the embeddings. This progression naturally led to increased research into the use of transformers for text classification and resulted in the development of 2 of the most prominent LMs: BERT [25] and GPT [26]. In these cases, transfer learning is employed to perform text classification. As previously discussed, scaling these PLMs led to the emergence of LLMs, which have revolutionized NLP tasks, with text classification being among the first.

### Research Objectives and Structure

The evolution of LMs, discussed in previous sections, from assistants to self-sufficient models capable of performing a wide range of NLP tasks has redefined how textual data are mined. Over the past few years, several systematic reviews have summarized and discussed research studies using LLMs in health care without focusing on specific tasks such as text classification [27-30]. In parallel, a few systematic reviews have explored current trends in health care text classification using NLP and machine learning techniques [31,32]. These reviews either addressed the general impact of LLMs on health care–related tasks or examined the full spectrum of machine learning–based approaches applied to health care text classification.

LLMs have transformed health care text classification, offering more cost-efficient and time-saving methodologies while potentially ensuring accurate categorizations. Consequently, a systematic review is needed to consolidate research studies that have leveraged LLMs for text classification in health care settings. This review examines literature published over the past 6 years, during which LLMs and PLMs were developed, used as-is, or fine-tuned to address various types of health care text classification. The collected studies are categorized, analyzed, and discussed, with the findings highlighting directions for future research.

This systematic review has 5 primary objectives: (1) to systematically identify, categorize, and analyze research studies that leverage LLMs for health care text classification across different health care text data types (ie, clinical notes, health care communications, and research/literature); (2) to examine and compare the methodological approaches employed, including prompt engineering, pretraining, fine-tuning, prompt-tuning, ensemble learning, data augmentation, and Retrieval-Augmented Generation (RAG), across different text classification types (ie, binary, multiclass, and multilabel); (3) to discuss the LLM types utilized (ie, BERT and variants, closed-source LLMs, open-source LLMs, and pretrained transformers) and their usage across diverse health care applications (ie, clinical decision support, research/literature analysis, public health and opinion analysis, patient safety and risk assessment, patient query analysis, information extraction, quality and equity, and other annotations); (4) to assess the ethical considerations and data-privacy preservation strategies implemented across studies; and (5) to identify existing gaps and limitations across data-, model-, methodology-, and ethics/privacy-related dimensions, and to propose future research directions for advancing LLM robustness in health care text classification through approach-based improvements, efficiency optimization, data-related contributions, and clinical practical implementation.

The remainder of this systematic review is organized as follows: The “Methods” section details the research methodology, which includes the paper collection process and inclusion/exclusion criteria. The “Results” section presents the results of the surveyed literature, focusing on text classification types, LLM-based methodologies, health care text types, and evaluation metrics. The “Discussion” section analyzes the results and highlights current research gaps and limitations. The “Future Research Directions” section explores future research directions. The “Conclusions” section summarizes this systematic review, focusing on health care text classification using LLMs.

## Methods

### Design

This systematic review follows the PRISMA (Preferred Reporting Items for Systematic Reviews and Meta-Analyses) guidelines (see Multimedia Appendix 1) for its research methodology [33].

### Search Strategy

This review considers 5 major databases for paper collection: Google Scholar, Scopus, ScienceDirect, Web of Science, and PubMed. It also includes references from the collected papers and another available literature search engine. The peer-reviewed articles and conference proceedings surveyed were published between 2018 and 2024. The paper search was conducted between March and September 2024. Keywords used include “large language models,” “llms,” “healthcare,” “medical,” “bio,” “biomedical,” “classification,” “text classification,” “text categorization,” and “sentiment analysis,” while excluding terms such as “image classification” and “survey.” In addition, only papers written in English were considered. This systematic review focuses on research studies that involve health care text categorization and evaluation using LLM-based approaches.

### Inclusion and Exclusion Criteria

The inclusion criteria for this systematic review are as follows: research studies (1) published in English; (2) published within the last 6 years; (3) published in peer-reviewed journals or conference proceedings; and (4) that utilized at least one PLM or LLM at any stage of text classification, regardless of type. Acceptable models include BERT and its variants, open-source research LLMs such as LLaMA 2, and closed-source models such as GPT-3.5/4. GPT-based models could be used either through application programming interface (API) requests or via ChatGPT, the inference model. The review considered various text classification types (ie, binary classification, multiclass classification, and multilabel classification) and applications (eg, clinical decision support, research/literature analysis, public health and opinion analysis, and patient query analysis). Health care textual data sources vary, for instance, from clinical notes and discharge summaries to patient comments and medical literature. Methodologically, the included studies may have used LLMs for direct classification, addressed class imbalance, applied data augmentation, fine-tuned models for specific health care text classification tasks, or pretrained models. Furthermore, research papers in which 1 or more health care datasets, among others, were used for evaluation are also included.

The exclusion criteria for the surveyed research papers include cases in which (1) the full text of the paper was inaccessible; (2) the study focuses on NLP tasks other than classification (eg, translation or extraction); (3) the evaluation approach relies on synthetically generated textual data; (4) the manuscript is a thesis, dissertation, workshop preface, editorial, letter to the editor, seminal contribution, comment, or review; (5) the paper primarily introduces a dataset or a library; (6) the paper deals solely with a non–health care application (eg, finance, legal, or social science) or focuses on LLM-generated text detection; (7) the classification is conducted on nontextual data (eg, images or time series); and (8) the study uses only traditional machine learning approaches without pretraining (ie, simple text vectorization, model training, and classification), without involving an LLM at any level.

## Results

### PRISMA Process

**Figure 3:**
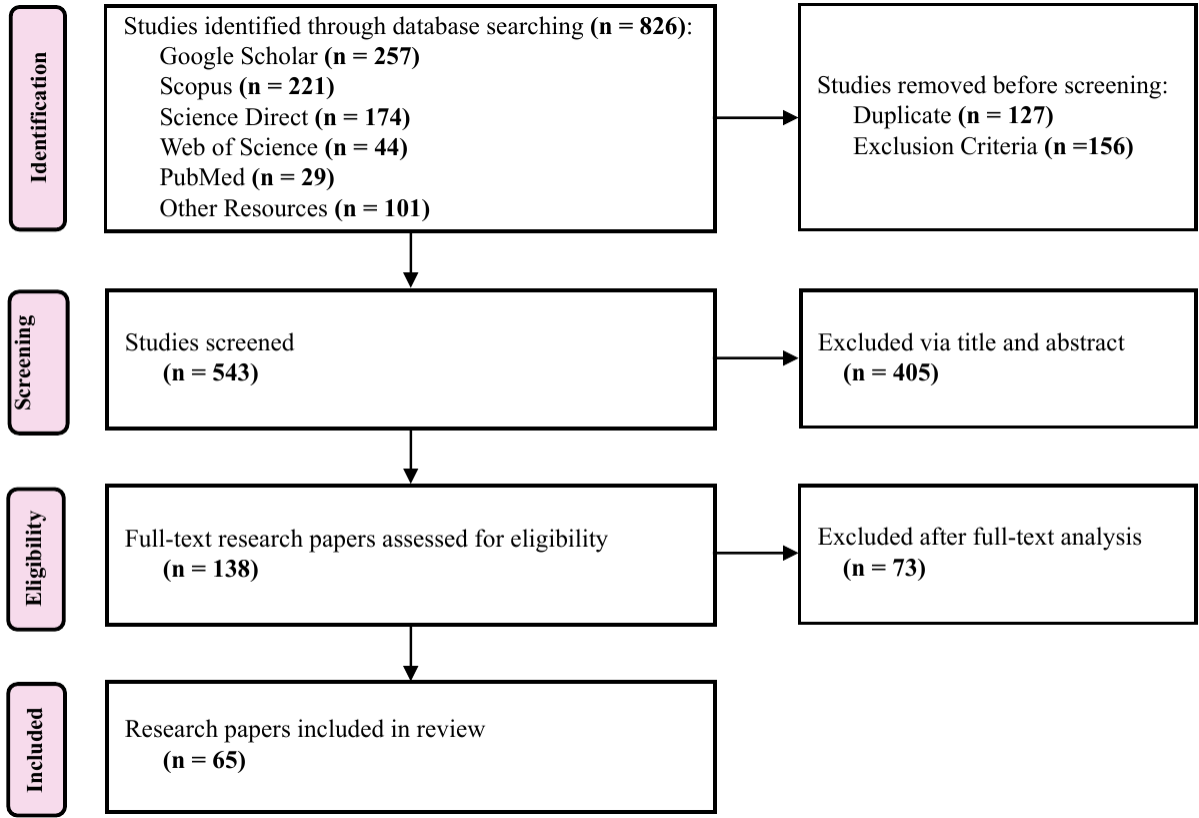
PRISMA (Preferred Reporting Items for Systematic Reviews and Meta-Analyses)-based search strategy.

At the “identification” stage, 826 research papers were collected, including 257 studies from Google Scholar, 221 from Scopus, 174 from ScienceDirect, 44 from Web of Science, 29 from PubMed, and 101 from other resources, which include references from research surveys and papers found through another available search engine. From this initial set, 127 papers were removed as duplicates, and 156 were excluded based on the aforementioned exclusion criteria. During the “screening” phase, 405 studies were excluded following the title and abstract review. Consequently, 138 research papers were retained and assessed for “eligibility” through full-text review. Of these, 73 studies were deemed ineligible, resulting in 65 research papers that were ultimately “included” and carefully examined in this systematic review, as shown in Figure 3.

### Overview of Selected Research Papers

**Figure 4:**
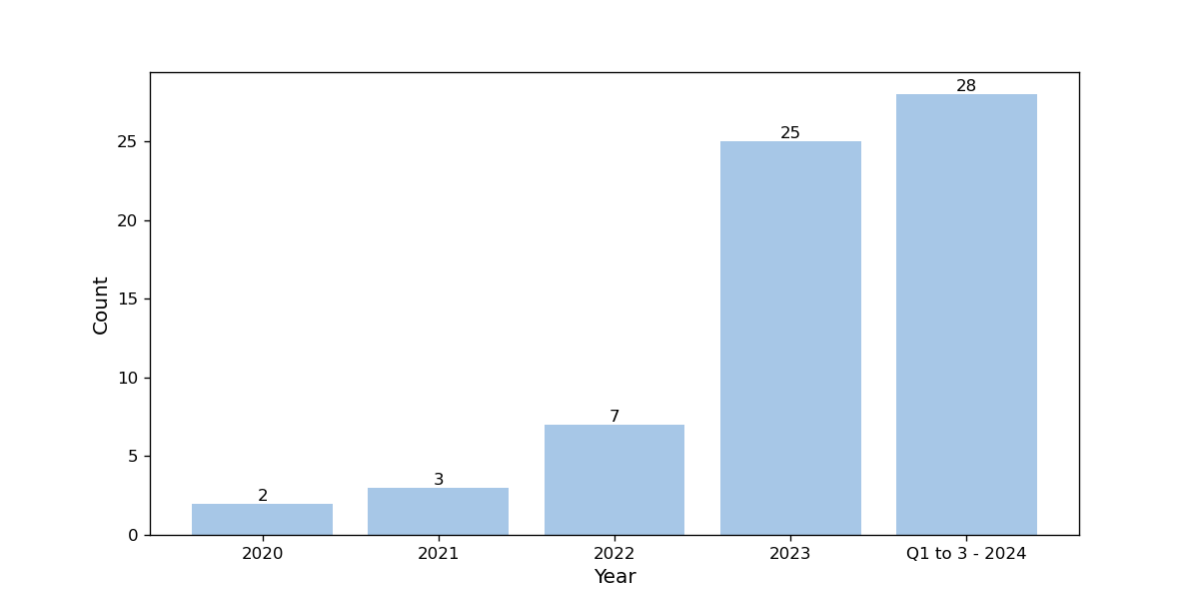
Eligible publications per year.

This systematic review includes 65 research papers published between 2018 and Q3 2024. This time frame was selected to capture the evolution and application of both PLMs and LLMs. For consistency with the literature, both model types are referred to as LLMs in this paper. Following the previously detailed exclusion criteria, the final publication years covered the period from 2020 to Q3 2024. Figure 4 shows the annual publication count over the last 5 years. This period is marked by a major shift in language modeling methods and applications. A significant increase is observed in the first 3 quarters of 2024, demonstrating an emerging trend in health care text classification in which LLMs are increasingly leveraged.

**Figure 5:**
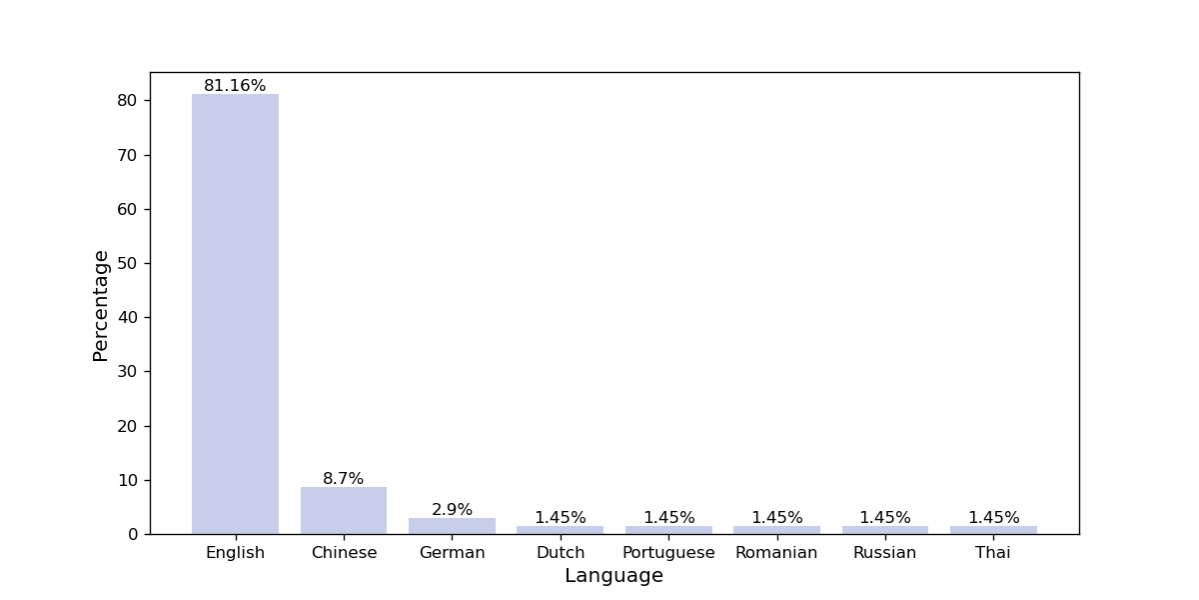
Eligible publications text data languages.

As the majority of available health care text data, regardless of type, are in English, more than 80% of the research studies deemed eligible covered entirely or partially English datasets, as shown in Figure 5. However, this is not the only reason for the observed text language distribution. Given the abundance of English text data, many popular LLMs (eg, BERT and GPT) were initially developed and trained in English, regardless of the application domain. Additionally, some researchers choose to translate health care textual data into English, particularly in the case of low-resource languages.

Health care text data used in classification studies can be categorized into 3 main types: *clinical notes, health care communications,* and *research/literature*. Clinical notes comprise the largest volume of data, primarily due to their continuous generation through daily medical procedures and health care operations. Health care communications represent the second-largest category, although their volume is significantly smaller than that of clinical notes. This category has expanded with the growth of social media usage and the increased adoption of patient satisfaction surveys by health care facilities. Research/literature documents constitute the smallest category, with a volume comparable to that of health care communications.

**Figure 6:**
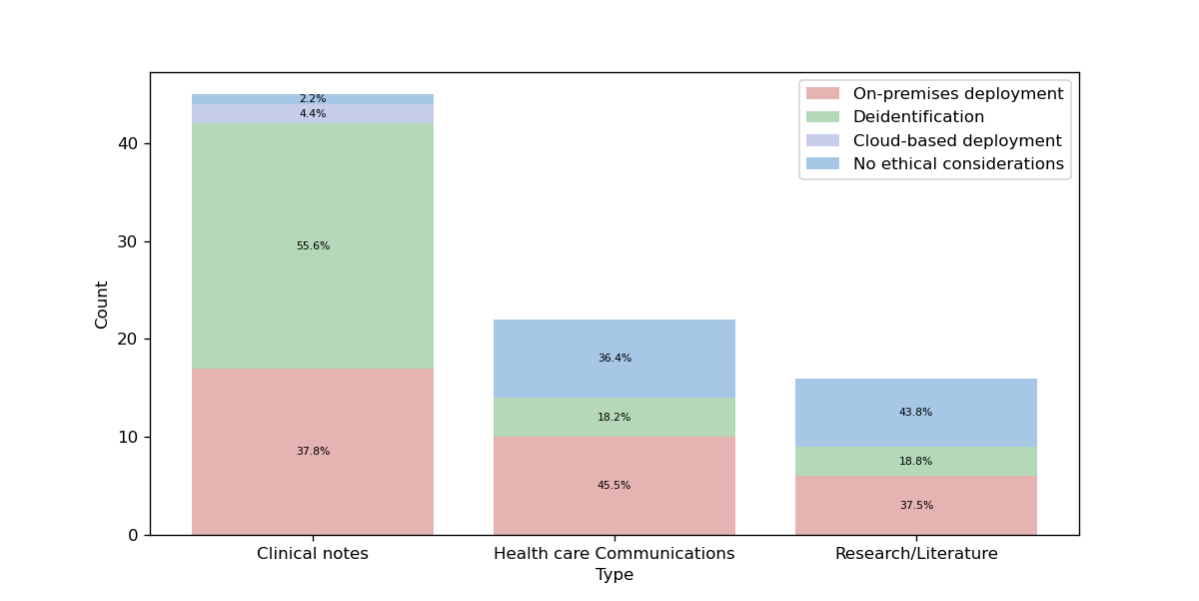
Health care text data categorization.

Figure 6 illustrates both the distribution of health care text data types and the ethical strategies employed for each category. The data reveal 3 main protective approaches: on-premises deployment, patient deidentification, and cloud-based deployment. The clinical notes category, which contains the most sensitive patient information, demonstrates the strongest ethical protocols. This category shows the highest rate of deidentification, closely followed by on-premises deployment, and is the only category that utilizes cloud-based deployment (via Azure Services). Notably, the clinical notes category has the lowest number of papers lacking explicit ethical considerations. By contrast, both the health care communications and research/literature categories frequently lack explicitly stated or clearly deduced ethical considerations, with research/literature showing the highest occurrence, followed by health care communications. Both categories exhibit similar deidentification rates of approximately 18%, while the remaining papers implement LLM-based on-premises deployment strategies.

On the one hand, Figures S1-S3 in Multimedia Appendix 2 illustrate the distribution of LLM types used for different text classification tasks in the eligible papers included in this systematic review. For binary classification, closed-source LLMs were most frequently used, followed closely by BERT (or variant) approaches, while open-source LLMs and pretrained transformers were used least frequently. In multilabel classification, BERT (or variant) models were predominant, followed by pretrained transformers, open-source LLMs, and finally closed-source LLMs. Bidirectional and Auto-Regressive Transformers (BART) made their first appearance in this category and were also utilized in multiclass classification. For multiclass classification, closed-source LLMs were the most common, followed by BERT (or variant), pretrained transformers, and open-source LLMs. Across all 3 classification types, BERT (or variant) models maintained a significant presence, likely due to their feasibility for local implementation, particularly through fine-tuning approaches. The analysis also revealed the popularity of closed-source LLMs, especially the GPT family, which were frequently employed in both multiclass and binary classification tasks. On the other hand, Figure S4 in Multimedia Appendix 2 categorizes the eligible papers by publication type, distinguishing between journal articles and conference proceedings. The analysis shows that these 2 categories have comparable representation in the literature.

### Taxonomy of Selected Research Papers

**Figure 7:**
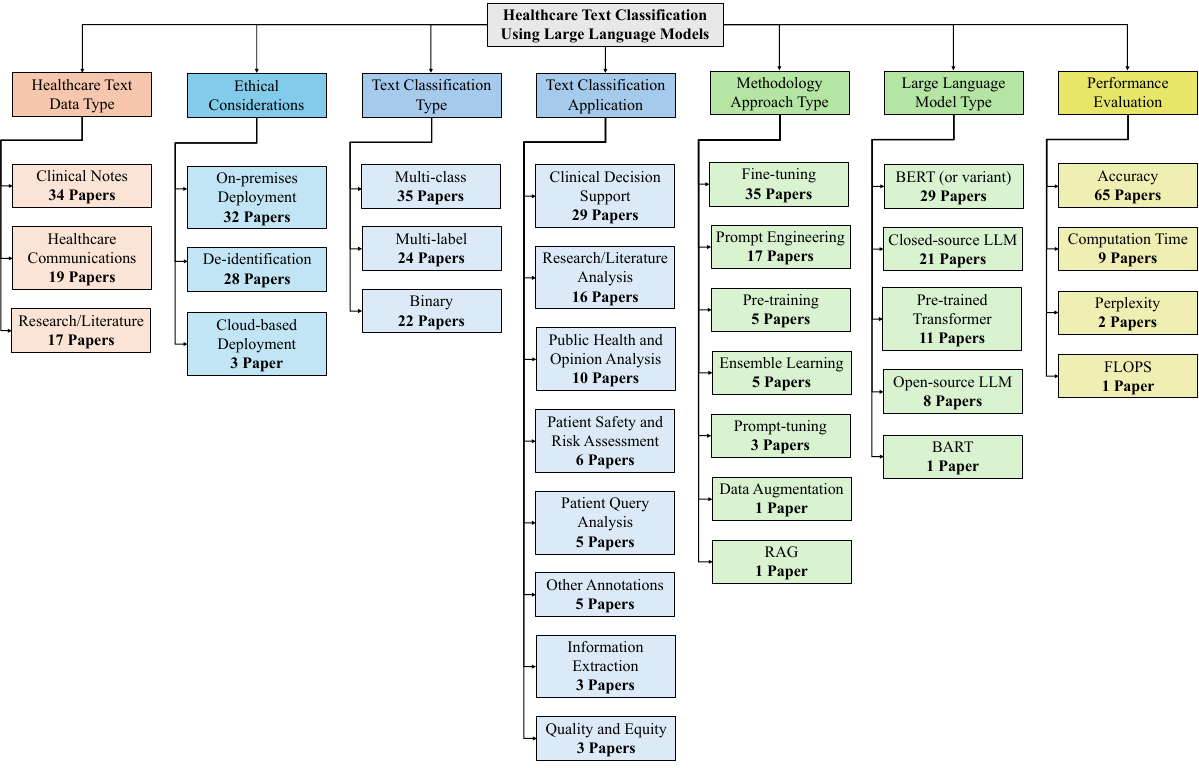
Eligible publications categorization. BERT: Bidirectional Encoder Representations from Transformers; FLOPS: Floating-Point Operations per Second; LLM: large language model; RAG: Retrieval-Augmented Generation.

The research papers resulting from the PRISMA eligibility phase are categorized according to 7 dimensions: health care text data type, ethical consideration, text classification type, text classification application, methodology approach type, LLM type, and performance evaluation. Figure 7 summarizes the categories associated with each dimension considered in this systematic review. It is worth noting that the LLM type reflects only the best-performing model(s) reported in each reviewed paper. In addition, the paper counts across categories do not necessarily sum to the total number of papers considered, as a single research study may include multiple classification tasks.

Health care text classification using LLMs can be comprehensively understood through the 7 previously mentioned, interconnected dimensions. These dimensions emerged from an iterative analysis of the 65 eligible research studies and provide a structured framework for understanding the current state of the field. The following subsections in the “Discussion” section explore each dimension in detail, examining how different combinations of health care text types, ethical approaches, classification types, applications, methodologies, LLM types, and evaluation metrics contribute to the overall landscape of LLM-based health care text classification.

## Discussion

### Overview

This section discusses and analyzes the eligible research papers based on the 7 previously introduced dimensions. Each dimension is described in detail and further examined according to the different categories it encompasses. As a result, research gaps and limitations are identified and highlighted.

### Health Care Text Data Type and Ethical Considerations

#### Data Types

The type of health care–related text data varies depending on the context in which it is collected. It encompasses a wide range of information sources, ranging from clinical notes to health care communications, literature, and research. Depending on the type of text used for classification, the impact on advances in medical research, patient care improvement, and clinical decision-making support may differ substantially. Ethical considerations also vary across research studies and can be broadly categorized into on-premises deployment, cloud-based deployment, and patient deidentification.

Clinical notes represent one type of health care–related text data that are experiencing extremely rapid growth. This type of data includes, for instance, progress notes, admission notes, discharge summaries, and treatment plans, and is generated by health care professionals during patient encounters or care delivery. Clinical notes are characterized by the use of medical jargon and abbreviations and are often inconsistent in formatting. Once digitally collected and stored, they can be classified for multiple purposes, such as serving as the basis for diagnosis decision-support tools. Depending on data availability and access, some researchers classified health care text using LLMs on clinical notes from specific hospitals, while others relied on publicly available clinical note datasets. Another frequently categorized type of health care text data that uses LLMs is health care communications. This category encompasses patient-generated text data, such as feedback from hospital surveys, comments collected from social media, or public opinions posted on various online health care platforms. Additionally, health care communications can include messages exchanged between hospital professionals, feedback provided to medical students by clinical mentors, and patient inquiries submitted through hospital portals. A third type of health care text data identified in the literature is research/literature, which includes health care research papers or medical blog articles that are classified. Figure 7 categorizes the health care text data types described above in the eligible studies. Table S1 in Multimedia Appendix 3 summarizes the research studies in which each health care text data type was used for LLM-based text classification, along with any ethical considerations employed.

#### Clinical Notes

Pathology reports have served as crucial data sources in clinical research. Sushil et al [34] analyzed breast cancer pathology reports from the University of California, San Francisco (UCSF) clinical data warehouse, which were manually labeled across 12 treatment-relevant categories. Bumgardner et al [35] investigated a large collection of surgical pathology reports from the University of Kentucky, focusing on cancer-related cases identified using International Classification of Diseases (ICD) condition codes. Chang et al [36] used pathology reports from The Cancer Genome Atlas project of the National Cancer Institute to extract pathologic tumor-node-metastasis staging information. This information was translated into 3 multiclass text classifications, and their evaluation relied on existing annotations. Additionally, the prompt-based text classification was validated using cancer-specific clinical reports, including breast invasive carcinoma and lung adenocarcinoma.

Radiology documentation has also been extensively studied across multiple research papers. Bressem et al [37] leveraged 3.8 million radiology reports, including chest radiographs and computed tomography (CT) scans, for pretraining and fine-tuning BERT models. A large subset of these reports was manually annotated for findings such as congestion, opacity, effusion, pneumothorax, and the presence of medical devices. This annotated set was divided into a larger subset for fine-tuning and a smaller subset for testing. Additionally, a small number of CT reports were used to evaluate the models’ performance on longer texts. Tan et al [38] examined CT reports from the National Cancer Centre Singapore across 4 cancer types, employing an 80-10-10 split for training, development, and testing. Uslu et al [39] utilized the Medical Information Mart for Intensive Care Chest X-Ray (MIMIC-CXR) dataset, focusing specifically on the “FINDINGS” section of radiology reports from chest X-rays. These reports were generated by radiologists interpreting chest radiographs of patients admitted to the emergency department (ED). The dataset contains detailed descriptions of radiological findings, classified into 14 distinct impressions, including 13 specific abnormalities (such as atelectasis, cardiomegaly, consolidation) and a “no finding“ category. Liu et al [40] evaluated various radiology-related text classification tasks (eg, sentence similarity, disease classification) using radiology reports from the MS-CXR-T (Multimodal Semantic Chest X-ray—Temporal), RadNLI (Radiology Natural Language Inference), and Chest ImaGenome datasets.

Discharge summaries have provided rich data for various analyses. Li et al [41] utilized MIMIC-III discharge summaries as part of their Silver dataset, annotated using LLaMA 65B. Alsentzer et al [42] examined obstetric-related discharge summaries from Mass General Brigham hospitals for postpartum hemorrhage classification. Wang et al [43] analyzed the “brief hospital course” sections of MIMIC-IV discharge summaries, which include key events, diagnostics, and treatments during hospitalization. Cui et al [44] studied temporal relationships in the n2c2 2012 challenge’s discharge summaries, which compile patient hospital stay information, including treatments and their temporal relationships. The task involved classifying whether treatments occurred during hospitalization or not.

Progress and visit notes have also provided insights into patient care patterns. Williams et al [45] analyzed ED physician notes, focusing on chief concerns and illness histories. Schneider et al [46] examined progress notes from a Brazilian tertiary hospital. Savage et al [47] studied MIMIC-III history and physical notes to investigate anticoagulant usage. Silverman et al [48] investigated outpatient clinical notes for inflammatory bowel disease from UCSF.

Specialty-specific documentation has also provided targeted clinical insights. Xie et al [49] classified seizure status in University of Pennsylvania Health System epilepsy notes as either “seizure-free” or “having recent seizures.” Guevara et al [50] analyzed radiotherapy and immunotherapy treatment notes from multiple institutions. The first dataset comprises clinical notes of patients with cancer who received radiotherapy at the Department of Radiation Oncology at Brigham and Women’s Hospital/Dana-Farber Cancer Institute in Boston, Massachusetts. At the same time, the second dataset comprises clinical notes of patients who received immunotherapy treatment and were not included in the first dataset. Chaichulee et al [51] examined Thai-English drug allergy records from Songklanagarind Hospital, covering 36 predefined symptom terms. Each record contains a free-text description of adverse drug reactions (ADRs) documented by health care professionals in a mixture of Thai and English.

Transcribed clinical interactions have provided unique perspectives on patient assessment. Ohse et al [52] analyzed GRID-HAMD-17 (GRID Hamilton Depression Rating Scale [17-item]) protocol interviews for depression classification, creating 5 distinct clusters. The interviews were conducted in German, but the transcriptions were translated into English for depression assessment. These transcriptions constituted the original dataset. Additionally, a clustered dataset was created to provide more context for text classification using LLMs. Each interview transcription was grouped into 5 clusters (ie, depression, anxiety, somatic, insomnia, and unimportant) based on the corresponding question. Balamurali and Chen [53] examined transcribed “Cookie Theft Picture” descriptions for Alzheimer disease (AD) assessment. These transcriptions were derived from speech produced by both patients with AD and cognitively normal individuals.

Administrative and coding documentation has supported various classification tasks. Yogarajan et al [54,55] utilized clinical notes for ICD-9 code prediction, while Kementchedjhieva and Chalkidis [56] focused on MIMIC-III discharge summaries for ICD-9 coding.

Specialized clinical datasets have enabled focused research objectives. Lehman et al [57] used the Clinical Language Inference for Patient Monitoring (CLIP) dataset, designed to capture key follow-up information in discharge summaries, as well as MEDNLI (Medical Natural Language Inference). Peng et al [58] leveraged multiple clinical note datasets for text classification. The clinical abbreviation disambiguation task was evaluated using an abbreviation dataset developed by the University of Minnesota, while the natural language inference (NLI) task used the MEDNLI benchmark dataset. Medication attribute filling was validated using the Contextualized Medication Event Dataset, and progress note understanding was assessed using the benchmark dataset developed for the 2022 n2c2 challenge (track 3), derived from MIMIC-III.

Critical care documentation has provided insights into acute care settings. Li et al [59] analyzed intensive care unit admission notes from MIMIC-acute kidney injury. Guevara et al [50] included critical care unit inpatient notes in their multiple-dataset study. Yogarajan et al [60] examined electronic intensive care unit program records alongside MIMIC-III data.

Longitudinal patient records have provided comprehensive views of patient care. Li et al [41] developed a Gold dataset from longitudinal electronic health record (EHR) notes of patients with AD. Pan et al [61] analyzed EHRs containing 11 implicit symptoms or diseases. Sivarajkumar and Wang [62] studied MIMIC-III patient notes for high-context phenotypes related to treatment and readmission risk. Yuan et al [63] used ClinicalTrials.gov to collect 6 stroke clinical trials, focusing on inclusion and exclusion criteria. Additionally, the UTHealth stroke patient database was used to retrieve patients’ EHRs containing diagnoses, procedures, and medications. Further comprehensive studies include those by Yang et al [64], McMaster et al [65], and Li et al [66], who utilized various MIMIC-III note types for model development and validation.

#### Health Care Communications

Health care communications research demonstrates diverse focus areas across several key domains. In mental health and psychological communications, multiple studies have examined different aspects. Lossio-Ventura et al [67] analyzed mental health during COVID-19 through web-based surveys, while Aldeen et al [68] and Ramteke and Khandelwal [69] focused on mental health manifestations in social media posts, particularly anxiety, depression, and stress. Xu et al [70] conducted a comprehensive analysis of mental health datasets from social media platforms, examining stress, depression, and suicidal ideation, complemented by Jiang et al’s [71] investigation of social anxiety and Farruque et al’s [72] analysis of depression-related tweets.

Within the framework of public health and vaccination communications, several researchers have explored vaccination-related discourse. Kim et al [73] examined human papillomavirus (HPV) vaccination messages across social media platforms, while Carneros-Prado et al [74], Bansal et al [75], and Ciobotaru and Dinu [76] focused on COVID-19 vaccination discussions, particularly analyzing public sentiment and concerns expressed on social media.

Clinical and medical services communications have been examined through various lenses. Wang et al [77] and Luo et al [78] utilized the KUAKE-QIC (Query Intent Classification) dataset, which compiles short texts representing patient inquiries and labels them into 11 intention classes for patient inquiry analysis. Ren et al [79] examined patient portal messages from clinical departments at Mayo Clinic, Shiju and He [80] analyzed drug reviews and medical conditions, and Kersting et al [81] investigated physician reviews and ratings. In medical education, Van Ostaeyen et al [82] uniquely focused on health care students’ ePortfolio feedback across different health care programs.

Disease-specific communications were represented by studies focusing on diabetes-related interactions, with both Ge et al [83] and Wu et al [84] analyzing diabetes-related questions and categorizing them into various classes. Finally, emotional and sentiment analysis in health care communications was explored by Gu et al [85], who examined 6-class sentiment expressions on Weibo; this analysis was also incorporated into Jiang et al’s [71] study of therapy-related sentiments.

#### Research/Literature

Research in disease-specific medical literature classification has covered various medical conditions. Shi et al [86] focused on cardiovascular diseases using the Ohsumed dataset, which implements single-label classification. Chen et al [87] developed a 3-class categorization system for diabetes mellitus papers, while Raja et al [88] categorized ocular disease literature into 19 categories. COVID-19 research has also been addressed, with Guo et al [89] analyzing treatment-related papers and Yang et al [90] developing a binary classification for SARS-CoV-2 and Nipah virus literature for drug discovery purposes.

In the scope of clinical and medical topics, Wang et al [29] tackled the classification of clinical trial screening criteria, developing 44 semantic categories through the CHIP-CTC (Chinese Health Information Processing – Clinical Trial Classification) dataset, which encompasses descriptive sentences. Sarkar et al [91] addressed the categorization of medical blog articles across 18 predefined topics, including headache, mental health, and heart health. Cancer research classification has been particularly significant, with multiple studies, including [92,93], utilizing the Hallmarks of Cancer dataset to classify cancer biology characteristics.

Other research studies have focused on different research classification tasks. Chen et al [94] developed a 3-tier advice classification system (no advice, weak advice, and strong advice) for medical research abstracts. Kementchedjhieva and Chalkidis [56] worked with the BIOASQ (Biomedical Semantic Question Answering) dataset, which consists of biomedical articles from PubMed and implements classification based on the Medical Subject Headings taxonomy. Qi et al [95] addressed industrial biomedical literature mining tasks, focusing on the recognition of specialized biomedical phrases. The data were acquired with inherent label noise due to crowdsourcing and labeling preferences. For testing, a subset of the data was relabeled and assumed to be clean.

General medical literature classification has been explored through various datasets. Yang et al [64] leveraged PubMed abstracts and Wikipedia articles for pretraining, while Schneider et al [46] utilized both PubMed and SciELO (Scientific Electronic Library Online) databases for fine-tuning. Gretz et al [96] contributed to this field by working with the Medical Abstracts dataset. Beţianu et al [97] and Luo et al [78] further expanded this research using PubMed datasets, with Luo et al [78] specifically incorporating multiple datasets, including BC7LitCovid (BioCreative VII Literature COVID-19 Track), for comprehensive biomedical literature classification.

#### Ethical Considerations

These research studies employed a wide range of data sources, from social media platforms to EHRs and literature databases, and utilized various categorization approaches, including binary, multiclass, and multilabel classifications. The research spans multiple languages and formats, reflecting the global and diverse nature of health care text classification. However, leveraging LLMs for these tasks in health care raises significant ethical concerns regarding patient privacy, data security, and potential algorithmic bias. The protection of sensitive health information is crucial under regulations that vary by country, such as the Health Insurance Portability and Accountability Act (HIPAA) in the United States. This is particularly important when using an LLM that requires API requests (eg, GPT-4o), where classification cannot be run locally, and when the text data consist of clinical notes containing patients’ protected health information, which must be detected and deidentified in advance. To address the challenges arising from the sensitive nature of health care textual data, researchers in the reviewed literature implemented various ethical safeguards and considerations. Some research papers conducted all their experiments locally, which is particularly convenient when fine-tuning BERT or its variants. Others anonymized textual data before providing them as input to the model or used already deidentified datasets (eg, MIMIC). By contrast, very few studies employed secure cloud-based deployments through services such as Microsoft Azure OpenAI. However, in many cases, ethical considerations were minimal, particularly for publicly available data, such as medical literature and some social media–based datasets.

Table S1 in Multimedia Appendix 3 details the specific approaches adopted by each study, which reflects varying ways for maintaining ethical awareness in the application of LLMs to health care text classification [98].

### LLM-Based Methodology Approach Type and Evaluation

#### Overview

This section categorizes the eligible research papers based on methodology type, as detailed in Figure 7. The methodology reported is the best-performing one for each study. Additionally, the text classification type and application are provided. The aim is to discuss and analyze these research studies. It is worth noting that some papers evaluated their methodology using multiple datasets; for this systematic review, only health care–related datasets are considered.

For each methodology category, 2 types of tables are included. These tables summarize each reviewed paper and provide detailed performance evaluation metrics. The focus is on accuracy (eg, ACC@1), *F*_1_-score (eg, micro), precision (eg, mean average precision), recall (eg, macro), and area under the curve score (eg, precision-recall, receiver operating characteristic). The reported metrics may refer to the overall performance of a dataset, a specific class, or a classification task.

#### Prompt Engineering

Prompt engineering is the practice of crafting input prompts that provide sufficient context to an LLM to maximize its performance on various NLP tasks. These tasks can range from text generation and translation to classification and summarization. This technique gained particular popularity with the launch of products such as ChatGPT and Claude. It involves designing the prompt’s format, wording, and structure to help the model better understand the task and improve the accuracy and efficiency of obtaining the desired output. Brown et al [2] demonstrated that carefully constructed prompts can significantly influence how an LLM interprets a task and produces results. They found that zero-shot learning (ie, where no input-output example pairs are provided in the prompt) and few-shot learning (ie, where typically 1-5 input-output example pairs are provided) benefit greatly from prompt engineering. By providing contextual clues or examples in the prompt, LLMs can generalize and perform tasks they were not explicitly trained on; this is also known as emergent behavior [8]. To help LLMs better align with human expectations, multiple prompt engineering techniques have been introduced in the literature. As discussed in the systematic review by Liu et al [40], for the specific task of text classification, these techniques can include cloze-prompts (eg, “The topic is [Z]”) or prefix-prompts (eg, “What is the sentiment?”). These techniques can be further enhanced through in-context learning (ie, including examples in the prompt) and prompt ensembling (ie, combining multiple prompts). The main goal is to formulate the classification task in a way that best leverages the LLMs’ pretrained knowledge. As a result, the LLM’s capabilities can be harnessed to perform text classification tasks without extensive additional pretraining or fine-tuning. Additionally, this technique efficiently eliminates the need for multiple specialized models, as a single LLM can adapt to various classification tasks through prompt design. For example, Schick and Schütze [99] introduced the pattern-exploiting training method, in which task-specific patterns or templates are used to rephrase the input text so that the LLM can better understand the classification task. In health care, the text to be classified can be particularly complex due to nuanced medical terminology and varying contexts. In this context, prompt engineering can guide the LLM and provide a scalable approach to implementing advanced text classification systems for health care applications, thereby facilitating more accurate and efficient health care decision-making.

The prompt engineering approach is the second most commonly used method in the literature covered in this systematic review, owing to its accessibility and efficiency. As summarized in Table S3 in Multimedia Appendix 3 and detailed further in Table S2 in Multimedia Appendix 3, the best-performing LLMs often belong to the GPT family. These general-purpose LLMs, trained on vast corpora, consistently demonstrate impressive capabilities across many NLP tasks, including text classification. In parallel, other studies have leveraged BARD, a variation of T5, or ClinicalBERT.

Sushil et al [34] used GPT-4 via Azure OpenAI Studio, ensuring HIPAA compliance, for zero-shot classification of breast cancer pathology reports. The methodology involved zero-shot classification using single prompts that requested all classification tasks simultaneously, with outputs structured in JSON format for automated evaluation. This study focused on extracting 12 key pathology features, including tumor characteristics, biomarkers, margin status, and lymph node involvement. This information can be useful for breast cancer diagnosis and treatment planning. Lossio-Ventura et al [67] used ChatGPT (based on GPT-3.5) for zero-shot sentiment analysis of COVID-19 survey responses. The method involved feeding individual documents to ChatGPT with the simple prompt, “What is the sentiment of the following sentence ‘x’?” where x was the text to be analyzed. As no examples were provided in the prompt, this constitutes a zero-shot setting. This study focused on sentiment analysis in free-text responses from 2 COVID-19 survey datasets: 1 from the National Institutes of Health and 1 from Stanford, capturing people’s experiences and attitudes during the pandemic lockdown. Shi et al [86] leveraged ChatGPT in combination with graphs, resulting in the proposed ChatGraph framework. ChatGPT was first used to refine the input text (ie, grammar correction and improved readability) and then to extract knowledge graphs as triplets (head entity, relation, and tail entity) using carefully designed prompts. These knowledge graphs were converted into text graphs, where words became nodes and relationships became edges, which were then used to train an interpretable linear classifier, the graph convolution network. Continuing the use of graphs, Chen et al [87] explored employing LLMs through 2 pipelines: LLMs-as-enhancers and LLMs-as-predictors. In the first pipeline, LLMs enhance node text attributes either at the feature level (by encoding text into embeddings) or at the text level (by augmenting text attributes), which are subsequently used by graph neural networks for predictions. The second pipeline directly uses LLMs to make predictions by converting graph structural information into natural language prompts. The LLM employed in this approach is ChatGPT (GPT-3.5-turbo-0613). Ohse et al [52] investigated the potential of 4 NLP models—BERT, LLaMA 2-13B, GPT-3.5, and GPT-4—to detect depression through clinical interviews. They tested 2 main strategies: zero-shot learning with the LLMs and a clustering approach, in which interview data were segmented into depression-relevant categories (depression, anxiety, somatic, and insomnia). GPT-4 achieved the highest accuracy for depression classification on the original (ie, nonclustered) data. Balamurali and Chen [53] assessed 3 LLM-based chatbots—ChatGPT-3.5, ChatGPT-4, and Bard—with the objective of detecting patients with Alzheimer dementia versus cognitively normal individuals using textual input from spontaneous speech recordings. The approach employed zero-shot learning with 2 levels of independent queries: The first consisted of a single direct question, “Could the following transcribed speech be from a cognitively normal or Alzheimer’s dementia subject?” The second used chain-of-thought (CoT) prompting to elicit more detailed information. The researchers analyzed recordings from the ADReSSo Challenge dataset, which were transcribed using Otter.ai. Aldeen et al [68] evaluated ChatGPT’s capabilities in data annotation tasks across 10 diverse datasets covering various subject areas and numbers of classes, including a Reddit-based mental health dataset. The approach involved testing different GPT models (GPT-3.5 and GPT-4), exploring various prompt strategies, and comparing results against human expert annotations. Liu et al [40] assessed GPT-4’s performance on text-based applications for radiology reports across various tasks, including sentence similarity classification (other annotations task 1), NLI (other annotations task 2), disease classification (clinical decision support task 1), and disease progression classification (clinical decision support task 2). The approach employed different prompting strategies, such as zero-shot, few-shot, CoT, and example selection, and compared GPT-4’s performance against state-of-the-art radiology-specific models. Among the datasets used for evaluation were MS-CXR-T and RadNLI. Kim et al [73] demonstrated a successful application of ChatGPT (GPT-3.5-turbo-0613) for analyzing vaccination sentiment in health care–related social media content, specifically focusing on HPV vaccination discussions. Their best-performing methodology involved collecting human-evaluated social media messages in short format (ie, Twitter) and long format (ie, Facebook) about HPV vaccination, inputting them into GPT-3.5, and generating 20 response instances per message to determine the message stance (antivaccination, provaccination, or neutral). This approach required no domain-specific pretraining or fine-tuning, making it an accessible and efficient tool for researchers analyzing public health discourse on social media. Carneros-Prado et al [74] compared GPT-3.5 with IBM Watson to analyze emotions and sentiments in COVID-19–related health care social media data. Their methodology involved processing COVID-19 tweets using a specific prompt engineering approach, in which GPT-3.5 was instructed to “rate the sentiment between -1 (negative) and 1 (positive)” and classify emotions into 5 categories: joy, sadness, fear, anger, and disgust. Without any specific training, GPT-3.5 demonstrated strong performance in detecting nuanced emotional expressions in health care–related tweets during the pandemic, particularly outperforming IBM Watson in recognizing irony and context-dependent sentiment in COVID-19 discussions. Williams et al [45] evaluated GPT-4’s ability to assess clinical acuity in ED settings using a dataset of adult ED visits from UCSF. The methodology involved creating 10,000 balanced pairs of ED visits with different Emergency Severity Index scores, in which each pair represented contrasting acuity levels, ranging from immediate to nonurgent. GPT-4 was prompted to analyze deidentified text—specifically the chief concern, history of presenting illness, and review of systems sections—from ED physician notes to determine which patient in each pair had higher acuity. In addition to performing well on this dataset, its results were comparable to a resident physician’s assessment in a 500-pair subsample. Sarkar et al [91] achieved the best performance for health care text classification by using ChatGPT-3.5 with a prompt-based methodology rather than an embedding-based approach. The authors designed a specific prompt structure that included a system setup explaining the task and defining the possible health-related topic categories (eg, “addiction,” “heart health,” “mental health”). The prompt template instructed the model to classify the input medical article into predefined categories and required responses in a specific JSON format: {“Topics”: [“List of topics”]}. Guo et al [89] developed a novel automated screening methodology using GPT-4 to evaluate titles and abstracts for inclusion or exclusion in clinical systematic reviews. The methodology involved crafting a specific prompt template that outlined the screening instructions, inclusion/exclusion criteria, and the abstract to be evaluated, requiring the model to respond with only “included” or “excluded.” They tested their approach on 6 review papers, covering studies on COVID-19 treatments, Raynaud syndrome, postoperative pain management, and clinical machine learning applications.

Alsentzer et al [42] leveraged Flan-T5-XXL to perform zero-shot classification of postpartum hemorrhage into 4 subtypes—uterine atony (tone), retained products of conception and placenta accreta spectrum (tissue), birth or surgical trauma (trauma), and coagulation abnormalities (thrombin)—from clinical discharge notes. When discharge summaries exceeded the model’s input length limit, they were split into 512-token chunks with a 128-token stride, predictions were generated for each chunk, and the results were subsequently aggregated. Raja et al [88] explored BART to automatically classify ophthalmology research papers. Five classification tasks were conducted, with the best-performing approach using zero-shot learning with BART to analyze titles and abstracts of ophthalmology articles from PubMed. This demonstrated BART’s ability to understand complex medical terminology and concepts without requiring additional training data or fine-tuning for specific medical domains. Sivarajkumar and Wang [62] introduced HealthPrompt, a zero-shot learning framework for clinical text classification using LLMs and prompt engineering, in which ClinicalBERT demonstrated the best performance. The framework processes clinical texts by first using a chunk encoder to split long medical documents into smaller segments, and then applying carefully designed prompt templates—either cloze or prefix prompts—to transform the input text into a classification. Chang et al [36] evaluated clinical LLMs (ie, Med42-70B) for automatically classifying cancer staging from pathology reports using a zero-shot chain-of-thought (ZS-CoT) prompting strategy. The methodology involved feeding unstructured pathology report text into Med42-70B along with a system prompt requesting a cancer staging review, followed by the instruction “Let’s think step by step,” which prompted the model to generate reasoning steps before producing the final tumor-node-metastasis classification.

The systematic comparison of the 17 prompt engineering–based research studies (ie, Tables S2 and S3 in Multimedia Appendix 3) reveals distinctive patterns in LLM selection, prompting strategies, and performance characteristics. Closed-source models, particularly the GPT family, dominated this approach, appearing in 12 of the 17 studies. Domain-specific models and pretrained transformers, such as Med42-70B and Flan-T5-XXL, demonstrated competitive performance, suggesting viable alternatives to general-purpose GPT models. Zero-shot approaches dominated the literature, reflecting prompt engineering’s primary advantage of requiring no training data, while chain-of-thought prompting emerged as a key alternative strategy. Performance patterns also reveal a clear relationship with task complexity: binary classifications tend to achieve higher accuracy, multiclass classifications show variable performance, and multilabel classifications appear least frequently. Application domains revealed strategic alignment, with clinical decision support and public health analysis showing strong results, while research and literature analysis demonstrated mixed performance. Prompt engineering’s key advantage—rapid deployment without fine-tuning—comes with significant trade-offs: API-based deployment raises privacy concerns, and operational costs can be substantial for high-volume applications.

#### Pretraining, Fine-Tuning, and Prompt-Tuning

##### Pretraining

Pretraining or further pretraining an LLM for health care text classification involves adapting the model’s language understanding to the specific health care domain. This approach enhances the model’s ability to process specialized terminology, abbreviations, and context found in health care texts, thereby improving classification performance. Pretraining typically occurs in 2 phases: the general transformer-based architecture (or existing LLM) is first trained on unlabeled curated data, followed by further pretraining on domain-specific datasets such as MIMIC-III or PubMed. Pretraining is usually followed by fine-tuning, which focuses on task-specific objectives, such as classifying patient diagnoses, symptoms, or treatments from clinical notes. This process enables the model to capture domain nuances, improving its generalization and reducing error rates in health care–specific tasks. Multiple existing health care–related LLMs result from further pretraining of general-purpose LLMs such as BERT. These models are referred to in this systematic review as BERT variants. For example, Lee et al [100] introduced BioBERT (Biomedical Bidirectional Encoder Representations from Transformers), a BERT model further pretrained on biomedical text from PubMed and PubMed Central, which demonstrated improved performance on named entity recognition and question-answering tasks in the medical domain. Similarly, Alsentzer et al [101] adapted BERT to clinical notes and released ClinicalBERT, which demonstrated significant improvements in health care NLP tasks, such as NLI, a classification task.

Yang et al [64] developed GatorTron-large, an 8.9-billion-parameter LLM specifically trained for health care applications using over 82 billion words of deidentified clinical text from UF Health’s EHRs, combined with additional medical text from PubMed, MIMIC-III, and Wikipedia. The model used a BERT-style architecture with 56 layers, 3584 hidden units, and 56 attention heads, trained using 2 self-supervised tasks that do not require prior manual labeling: masked language modeling and sentence-order prediction. GatorTron-large demonstrated superior performance on various clinical NLP tasks critical for health care applications, including NLI, which determines logical relationships between clinical statements. The model’s extensive training on real clinical narratives from diverse health care settings—including inpatient departments, outpatient departments, and EDs—enabled it to better understand health care text data. McMaster et al [65] leveraged DeBERTa (Decoding-enhanced Bidirectional Encoder Representations from Transformers) to automatically detect ADRs in hospital discharge summaries. Their best-performing model, MeDeBERTa, used a 2-stage training process. First, the base DeBERTa model was further pretrained on a large set of unlabeled clinical documents from the studied hospital to adapt it to the institutional context. Then, fine-tuning was performed on this pretrained model using annotated discharge summaries enriched with validated ADR cases, enabling the model to distinguish true ADRs from other drug-related adverse events. Li et al [59] developed 2 clinical domain–specific LMs, Clinical-Longformer and Clinical-BigBird, designed to handle long clinical texts from EHRs. Starting with pretrained Longformer and BigBird models, additional pretraining was performed using clinical notes from MIMIC-III. This pretraining extended the models’ maximum input sequence length from 512 to 4096 tokens, enabling better capture of long-term dependencies in clinical narratives. Both models employed sparse attention mechanisms that combine sliding windows and global attention to reduce computational costs. The pretrained LLMs were evaluated on various NLP tasks following their respective fine-tuning. Clinical-Longformer outperformed conventional LLMs, such as ClinicalBERT. Bressem et al [37] leveraged BERT to classify radiology text reports, specifically focusing on chest radiographs. The authors additionally pretrained the German BERT base model on radiology reports from their institution, resulting in the development of RAD-BERT. Subsequently, this LLM was fine-tuned (FT RAD-BERT) to classify various radiological findings, such as congestion, effusion, consolidation, and pneumothorax. Blinov et al [102] focused on classifying clinical diagnoses from EHR text data using a modified BERT-based model, RuPool-BERT, adapted for Russian. The proposed methodology involved 2 key ideas: first, modifying RuBERT’s architecture (a standard BERT model adapted for Russian) by concatenating the classification token output with 2 additional components—max and mean pooling over the last encoder states—before passing it through the final classification layer; and second, developing a domain-specific version, RuEHR-BERT, by pretraining BERT on medical records using masked language modeling and a custom medical tokenizer trained on the same data. The resulting LLMs, RuPool-BERT and RuEHR-BERT, outperformed baseline models, with RuPool-BERT showing a slight edge in performance. It is worth noting that further pretraining of an LM is typically followed by fine-tuning the resulting model.

##### Fine-Tuning

Fine-tuning LLMs involves adapting pretrained models to specific tasks or domains by training them on additional data. In some cases, this process resembles the conventional supervised learning paradigm, as labeled data are required. Fine-tuning builds on the LLM’s knowledge acquired during pretraining while narrowing its focus to the nuances of a particular application. Fine-tuning is particularly valuable in scenarios where the general-purpose language capabilities of LLMs require refinement to capture domain-specific jargon, structure, or patterns, as is often the case in health care. The primary benefit of fine-tuning LLMs for health care is their ability to improve classification performance on complex text inputs without the need for massive amounts of labeled data from scratch. Additionally, fine-tuning provides greater flexibility for health care–specific needs, such as identifying diagnoses and treatments or extracting structured information from unstructured text. This adaptation is achieved by updating the LLM’s parameters with task-specific data. Several techniques are used for fine-tuning. For example, standard fine-tuning is the traditional method that updates all of the LLM’s parameters for the downstream task [25]. It is highly effective but computationally expensive and requires a large amount of labeled data. There are also more efficient methods, such as parameter-efficient fine-tuning (PEFT). These approaches selectively fine-tune a subset of the model’s parameters, reducing computational costs while maintaining performance. Adapter layers and Low-Rank Adaptation (LoRA) are notable PEFT techniques. Recently, LoRA has gained particular attention and has been widely adopted in resource-constrained environments [103]. Each fine-tuning approach aims to balance efficiency, generalization, and computational cost based on the specific use case and available resources. As a result, the model retains the general linguistic understanding gained during pretraining while adapting to the particularities of health care terminology, abbreviations, and context-sensitive phrases.

Fine-tuning emerged as the most commonly used LLM-based approach in the reviewed literature, outperforming pretraining and prompt engineering in 36 of 65 research studies. Its popularity stems from its ability to balance computational efficiency and task-specific performance, leveraging pretrained model foundations while adapting to health care data and text classification tasks. This approach leads to higher performance without the need to train a model architecture from scratch or rely on potentially weaker prompt-based methods. Moreover, including early PLMs (eg, BERT and GPT-2) in this systematic review highlights that researchers had ample time to experiment with these models. Their smaller size also made them well-suited for fine-tuning, given the lower computational requirements.

Ohse et al [52] evaluated LLMs for depression detection from clinical interview transcripts, with GPT-3.5 (after fine-tuning) achieving the highest *F*_1_-score. The methodology involved collecting interviews from participants using the GRID-HAMD-17 protocol, with participants also completing the 8-item Patient Health Questionnaire (PHQ-8) as a baseline measure of depression. The interviews were recorded, transcribed from German to English using the Whisper model, and then segmented into 4 clinically relevant clusters: depression, anxiety, somatic, and insomnia. GPT-3.5 was fine-tuned using 12 interview samples with the highest error rates in initial testing, training the model to predict PHQ-8 scores from the clustered interview text. This fine-tuned approach significantly outperformed zero-shot implementations of other models, including GPT-4 and LLaMA 2, in accurately detecting depression from clinical interview transcripts. Schneider et al [46] developed GPT2-Bio-Pt, a Portuguese LM specifically designed for biomedical and clinical text analysis, by fine-tuning an existing Portuguese GPT-2 model (GPorTuguese-2) on a large corpus of biomedical literature, including PubMed and SciELO databases. The model was evaluated on a health care–specific task: detecting patient fall events in deidentified clinical progress notes from a Brazilian hospital. As a result, the fine-tuned GPT2-Bio-Pt achieved the best performance. It is worth noting that GPT-2 is not a fully open-source LLM and is therefore considered a closed-source model in this review.

Xu et al [70] presented Mental-Alpaca and Mental-FLAN (Fine-Tuned Language Net)-T5, 2 instruction fine-tuned LLMs for mental health analysis using social media text data. The best-performing approach involved instruction fine-tuning the base LLMs (Alpaca and FLAN-T5) simultaneously on multiple mental health datasets from social media platforms, primarily Reddit, covering various conditions such as stress, depression, and suicide risk. The fine-tuning process combined multiple datasets and tasks into a single training iteration, allowing the models to learn various mental health prediction tasks concurrently. This approach enabled the models to outperform much larger models, such as GPT-3.5 and GPT-4, on tasks including the detection of depression, stress, and suicide risk from user-generated text. The resulting 2 LLM-based approaches achieved higher performance across multiple tasks and datasets compared with other models. Guevara et al [50] evaluated different configurations of fine-tuned FLAN-T5 models to automatically extract Social Determinants of Health (SDoH) from clinical notes in EHRs. For this purpose, the PEFT method, LoRA, was employed. The models were trained to identify 6 key SDoH categories: employment status, housing issues, transportation issues, parental status, relationship status, and social support. Using a dataset of clinical notes from patients with cancer, the researchers experimented with different model architectures and synthetic data augmentation approaches. The addition of synthetic data, generated using GPT-3.5, proved particularly beneficial for smaller FLAN-T5 models and for rare SDoH categories with limited training examples. Gretz et al [96] achieved the best performance for health care text classification by fine-tuning FLAN-T5-XXL using a leave-one-fold-out setup on health care datasets. LoRA was employed for efficiency, and training was conducted for 3 epochs with early stopping based on development set performance. Kementchedjhieva and Chalkidis [56] proposed T5Enc, a T5 LM fine-tuned in a nonautoregressive fashion. The model was tasked with assigning multiple Medical Subject Headings terms to biomedical articles and ICD-9 diagnostic codes to clinical notes. Fine-tuning was performed using the Adafactor optimizer with a fixed learning rate following a warm-up for 1 epoch. The model was trained to minimize cross-entropy loss on the binary classification task for each medical label.

Li et al [66] developed LlamaCare, a clinical domain–adapted LM created by instruction fine-tuning LLaMA 2 (7B chat version) on health care text data. Their methodology involved a 2-step process. First, GPT-4 was used to generate diverse clinical instructions based on seed prompts for different medical service types (eg, radiology, respiratory, rehabilitation). Second, corresponding input-output pairs were extracted from the MIMIC-III clinical database, which contains various medical notes, including discharge summaries, electrocardiogram reports, and nursing notes. The model was fine-tuned using LoRA to efficiently adapt LLaMA 2 to clinical tasks while minimizing computational resources. The proposed approach was evaluated on various tasks, among which mortality prediction, length of stay prediction, diagnoses prediction, and procedures prediction can be categorized as text classification tasks. Bumgardner et al [35] demonstrated the successful application of fine-tuned LLaMA models to extract structured condition codes from pathology reports in a health care setting, with the larger 13 billion parameter version showing superior overall performance. The researchers trained Path-LLaMA using surgical pathology reports containing gross descriptions, final written diagnoses, and ICD condition codes from clinical workflows at the University of Kentucky. The training data were formatted as instruction-based conversations in JSON format, in which each pathology case’s text was concatenated into a single input field, with the associated ICD-9 codes provided as the model response. The researchers focused specifically on cases with cancer-related codes. Wang et al [43] developed DRG (Diagnosis-Related Group)-LLaMA, a fine-tuned version of the LLaMA LM, to predict diagnosis-related groups from clinical discharge summaries in the MIMIC-IV dataset. The authors extracted the “brief hospital course” section from discharge summaries and fine-tuned LLaMA using LoRA. The best-performing model used a 13-billion-parameter version of LLaMA with a maximum input context length of 1024 tokens.

Lehman et al [57] evaluated multiple LMs on 3 clinical tasks using health care text data from EHRs. Two of these tasks are classification-based: NLI and the identification of follow-up information in discharge summaries. The best-performing approach utilized BioClinRoBERTa with task-specific fine-tuning. This model achieved superior performance compared with larger general-purpose models, including GPT-3, demonstrating that domain-specific pretraining on clinical text was more valuable than model size alone for this health care application. As previously discussed in the “Prompt Engineering” section, Raja et al [88] developed a text classification framework for ophthalmology research papers using BART in a zero-shot learning approach. However, for clinical study subclass grouping, where the initial BART model showed lower performance, they fine-tuned BioBERT, which performed better. Savage et al [47] developed and validated a fine-tuned BioMed-RoBERTa model to screen patients for appropriate best practice alerts in EHRs, specifically focusing on identifying patients who should receive deep vein thrombosis prophylaxis. The model was fine-tuned using history and physical notes from the MIMIC-III database, restricted to patients who did not receive anticoagulation at admission. Two physicians labeled these notes as either indicative of active bleeding or no active bleeding. The fine-tuned model was trained to perform binary classification of clinical notes to identify patients without active bleeding who would be appropriate for thromboembolism prophylaxis alerts. A development set of notes was used to optimize hyperparameters, and a separate test set was used for evaluation. As a result of token limits, patient notes were truncated to 2000 characters. Shiju and He [80] demonstrated that Bio_ClinicalBERT best classified drug reviews from Drugs.com when fine-tuned on a binary classification task. Their methodology involved fine-tuning Bio_ClinicalBERT on a dataset of drug reviews using a binary classification approach in which ratings of 8 or higher were considered “above average” and ratings under 8 were considered “below average.” Xie et al [49] developed an epilepsy-specific LLM approach to identify seizure outcomes from clinical notes by fine-tuning Clinical_BERT using manually annotated epileptologist notes. The model was trained to classify each clinical visit note as either “seizure-free” (no seizures since the last visit or within the past year) or “having recent seizures.” The fine-tuning process involved a plurality voting system, in which model predictions were repeated 5 times using different random seeds. Chen et al [92] proposed LitMC-BERT, a transformer-based multilabel classification model specifically designed for biomedical literature classification. The architecture uses BioBERT as its shared transformer backbone and introduces 2 novel components: label-specific modules, which capture unique features for each medical topic (eg, treatment, diagnosis, prevention) through multihead self-attention, and a label pair module, which models relationships between co-occurring medical topics through coattention mechanisms. The training process involves multitask learning, in which the model simultaneously learns to predict labels and their co-occurrences, followed by fine-tuning only the “label” module while keeping other components frozen. This approach demonstrated effectiveness in the medical domain by outperforming existing methods in classifying both COVID-19 literature and cancer research papers (ie, Hallmarks of Cancer). Cui et al [44] focused on classifying temporal information about medical treatments in hospital discharge summaries, specifically determining whether treatments occurred during hospitalization periods. The best-performing approach used BERT with traditional fine-tuning. The methodology involved preprocessing clinical text by extracting relevant sentences using a window-based approach (3 sentences before and 2 after the target treatment mention), along with admission and discharge dates. The fine-tuning process adapted BERT’s pretrained weights to learn temporal relationships between medical treatments and hospitalization periods, enabling health care professionals to automatically track when specific medical interventions occurred during a patient’s hospital stay. Van Ostaeyen et al [82] fine-tuned RobBERT, a Dutch BERT-based LM, to automatically analyze written feedback in health care education settings. Using a dataset of labeled feedback comments, split into sentences and collected from 5 health care educational programs (ie, specialistic medicine, general practice, midwifery, speech therapy, and occupational therapy), the authors trained multiclass, multilabel classification models to identify both feedback quality criteria and Canadian Medical Education Directions for Specialists (CanMEDS) roles in the health care text. The fine-tuning process involved tokenizing the health care feedback data, padding or truncating sentences to a fixed length of 512 tokens, and optimizing hyperparameters through 5 optimization runs on a development dataset. Ren et al [79] demonstrated the successful application of fine-tuned BERTweet, a RoBERTa-based LM pretrained on Twitter data, for classifying patient portal messages in health care settings. The model was fine-tuned on 2239 annotated patient portal messages from 3 clinical departments (ie, cardiology, gastroenterology, and dermatology) to classify messages into 4 categories: active symptoms, prescriptions, logistics, and update/other. The fine-tuning process involved training for 4 epochs using the Adam optimizer and the cross-entropy loss function. Chen et al [94] evaluated GPT-4 using few-shot prompts with CoT reasoning to address biomedical text classification. They analyzed medical research literature to categorize health advice sentences into “no advice,” “weak advice,” and “strong advice” across different sections of medical papers (in the discussion section, structured abstracts, and unstructured abstracts). In parallel, BioBERT was fine-tuned in a standard supervised manner and outperformed the aforementioned approach. Silverman et al [48] used 2 variants of UCSF-BERT, a clinical LM pretrained on millions of clinical notes from UCSF’s EHRs, to identify serious adverse events (SAEs) from inflammatory bowel disease clinical notes. The base hierarchical model (H-UCSF-BERT) can process longer sequences of up to 2560 tokens by combining chunk representations through an additional transformer layer and performed best for detecting whether a medication was mentioned or given before a hospitalization event (task 1). A modified version (H-UCSF-BERT + only nearby SAEs), which restricted the analysis to SAEs mentioned within a 2-sentence window of hospitalization events, achieved the best results for identifying whether a hospitalization was caused by or related to an adverse event (task 2) and whether a medication was given before a hospitalization that was caused by an adverse event (task 3). Chen et al [93] used a standard supervised fine-tuning approach on task-specific biomedical datasets from the Biomedical Language Understanding & Reasoning Benchmark (BLURB), which includes literature classification tasks. The model evaluated was BioLinkBERT-Large. Beţianu et al [97] proposed DALLMi for effective domain adaptation in health care text classification using BERT as the base LLM. DALLMi used a semisupervised fine-tuning technique that combines 3 key components: a label-balanced sampling strategy that ensures at least one positive sample per label in each batch; a novel variational loss function that leverages both labeled and unlabeled medical text data; and a MixUp regularization technique that performs interpolation at the word embedding level of BERT to generate synthetic training samples. Farruque et al’s [72] developed a semisupervised learning approach using Mental-BERT to detect depression symptoms from social media text data. The methodology first involved fine-tuning Mental-BERT on a clinician-annotated dataset of depression-related tweets, in which tweets were labeled with specific depression symptoms according to established guidelines. The researchers then employed an iterative data-harvesting approach, in which this initially fine-tuned model, coupled with a zero-shot learning model (USE-SE-SSToT), was used to automatically label additional depression-related tweets from a curated repository of posts from self-disclosed depressed users. These newly labeled tweets were then used to retrain the model, creating an expanded training dataset that maintains the clinical distribution of depression symptoms. The study evaluated 2 classification tasks: depression symptoms detection and depression post detection. Pan et al [61] presented Feature-Level Attention for Multilabel Classification on BERT (FAMLC-BERT), a fine-tuned BERT-based model designed to predict multiple medical diagnoses from unstructured clinical free text in EHRs. The methodology involved fine-tuning the BERT base model with a feature-level attention mechanism that captures semantic features from different BERT encoder layers. This attention mechanism assigns different weights to the [CLS] token embeddings from each of BERT’s 12 encoder layers, allowing the model to focus on the most relevant semantic features for disease prediction. The fine-tuning process used binary cross-entropy loss and the Adam optimizer, with the input text preprocessed to handle Chinese characters and to standardize special tokens such as dates and numbers. Bansal et al [75] used a fine-tuned DeBERTa large model to classify health care–related social media posts, specifically tweets expressing concerns about COVID-19 vaccines. The best-performing model was fine-tuned using the CAVES dataset, which contains tweets. This application is particularly relevant to health care because it enables the automatic categorization of vaccine-related concerns into 12 distinct categories, including side effects, efficacy, rushed development, and religious reasons. This capability allows public health officials to better monitor and address vaccine concerns in a timely manner. Uslu et al [39] focused on using a BERT variant to classify chest X-ray radiology reports from the MIMIC-CXR dataset into 14 distinct medical findings. The best-performing approach utilized CXR-BERT-GENERAL, which was fine-tuned on the “FINDINGS” section of radiology reports using binary cross-entropy loss and the Adam optimizer. Qi et al [95] developed SaFER, a 2-stage fine-tuning framework for BERT that was successfully applied to biomedical literature mining tasks. The fine-tuning approach consists of 2 key stages. First, they fine-tuned BERT using a label-agnostic early stopping strategy based on local intrinsic dimensionality scores to determine the optimal stopping point before overfitting occurs. Second, they implemented contrastive learning with a projection head alongside the classifier, using SimCSE (Simple Contrastive Sentence Embeddings) to generate positive pairs and applying structural loss to maintain consistency between the classifier and the projection outputs. The proposed approach was evaluated on 2 tasks related to research/literature analysis, although these tasks were not explicitly described. Ciobotaru and Dinu [76] used a fine-tuned Romanian BERT model to analyze public sentiment around COVID-19 vaccination in Romanian tweets. Specifically, they fine-tuned the Romanian-BERT-cased model by adding a linear layer with a softmax activation on top of the base model and trained it for 10 epochs using cross-entropy loss and the AdamW optimizer. The model was trained on the SART (Sustained Attention to Response Task) dataset, which contained tweets. Kersting et al [81] developed an efficient approach for analyzing German physician reviews using XLM-RoBERTa-large (Cross-lingual Language Model – RoBERTa [Large]), which was fine-tuned for aspect-based sentiment analysis. The authors combined 3 traditionally separate steps (ie, aspect term extraction, aspect category classification, and aspect polarity classification) into a single model through token classification. The model was fine-tuned on annotated physician review data to simultaneously identify relevant aspects (eg, physician friendliness, competence, or waiting times), classify them into predefined categories, and determine their sentiment polarity. The methodology was evaluated on 4 datasets (ie, A, B, C, and D). Yogarajan et al [54] explored the use of fine-tuned LLMs for automated medical code prediction from EHRs. PubMedBERT and BioMed-RoBERTa-base achieved the best results among the LLM approaches tested. The methodology involved fine-tuning these pretrained models on medical text data from 2 distinct EHR databases (MIMIC-III and electronic intensive care unit) for multilabel classification of ICD-9 codes. Specifically, the researchers used standard fine-tuning across all layers without freezing, employing the Adam optimizer.

Ge et al [83] developed a fine-tuned LLM approach to classify diabetes-related patient questions into 6 categories (ie, diagnosis, treatment, common knowledge, healthy lifestyle, epidemiology, and others). Their methodology leveraged the Baichuan2-13B model and employed a 2-stage fine-tuning process. First, the model was fine-tuned on a broader medical dataset (PromptCBLUE) using the LoRA technique, followed by transfer learning on the diabetes-specific question dataset. Tan et al [38] developed a fine-tuned LLM-based approach to automatically classify cancer disease responses from radiology reports. Specifically, they used the GatorTron transformer model and fine-tuned it on CT reports from patients with cancer, which were manually annotated into 4 categories: no evidence of disease, partial response, stable disease, or progressive disease. The best-performing approach combined traditional fine-tuning with a novel data augmentation technique using sentence permutation, in which synthetic training examples were generated by randomly reordering sentences in the radiology reports while maintaining the same label. Yogarajan et al [55] achieved the best results in automatic ICD-9 code prediction from clinical notes using TransformerXL (Transformer with Extra-Long Context), an LM capable of handling longer sequences. Fine-tuning was performed using binary cross-entropy loss and the Adam optimizer, without freezing any parameters. Yogarajan et al [60] reported that the best-performing LLM-based approach for predicting shielding among patients with COVID-19 from EHRs also used TransformerXL. This task involved identifying patients who are clinically extremely vulnerable to coronavirus based on their medical conditions. The methodology included fine-tuning TransformerXL on medical text from the MIMIC-III dataset, which contains discharge summaries. The model was fine-tuned end to end across all layers using the Adam optimizer, with a sigmoid activation function for multilabel classification of ICD-9 medical codes to indicate high COVID-19 risk. Luo et al [78] proposed Taiyi, a bilingual fine-tuned LLM for biomedical NLP tasks. The methodology involved fine-tuning Qwen-7B-base on a comprehensive collection of 140 biomedical text datasets (102 English and 38 Chinese) spanning over 10 task types, including text classification. The key innovation in their fine-tuning approach was a 2-stage supervised instruction fine-tuning strategy using QLoRA (Quantized LoRA): first, fine-tuning on nongeneration tasks, such as information extraction and tasks with smaller datasets; then, combining all data for a second stage of fine-tuning that included question-answering and dialogue tasks. The training data were carefully curated and standardized using consistent schema templates, with particular attention paid to selecting high-quality health care datasets.

##### Prompt-Tuning

Prompt-tuning bridges the gap between prompt engineering and fine-tuning. Unlike prompt engineering, which involves manually designing prompts to maximize an LLM’s efficiency and achieve desired outputs, prompt-tuning uses trainable soft prompts [104]. In this approach, instead of manually crafting prompts as in traditional prompt engineering, the prompts are represented as learnable vectors. These vectors are trained alongside the model on a specific task. These soft prompts guide the LLM in performing health care text classification without requiring extensive human-designed inputs, unlike traditional prompt engineering, which relies heavily on manual intervention. By contrast, fine-tuning adjusts a large number of the LLM’s parameters, often demanding significant computational resources and large labeled datasets. Prompt-tuning, however, updates only the prompt embeddings while keeping the rest of the LLM frozen. Thus, prompt-tuning strikes a balance between the resource-intensive nature of fine-tuning and the simplicity of prompt engineering, enabling task-specific adaptability in health care contexts with minimal computational cost.

Wang et al [29] used ERNIE (Enhanced Representation through Knowledge Integration – Health)-Health, a discriminative LLM specialized for health care, combined with prompt-tuning to classify health care text data. Instead of traditional fine-tuning, which adds extra classification layers, their best-performing method reformulates the classification task as a mask prediction task. Specifically, medical text inputs are wrapped into natural language templates where category labels are replaced by [UNK] tokens. ERNIE-Health’s multitoken selection pretraining task is then leveraged to predict the correct category label from a set of candidate options. The prompt-tuning approach proved particularly effective for the smaller medical dataset (KUAKE-QIC), outperforming traditional fine-tuning by leveraging ERNIE-Health’s pretrained medical domain knowledge without requiring additional parameters or extensive training data. Peng et al [58] developed a unified approach to clinical text classification using GatorTronGPT, a GPT-3 model further trained on a large corpus of clinical text. A key element of this methodology was the use of soft prompting with a frozen LLM, where the model’s original parameters remained unchanged and only the trainable vector prompts were optimized during fine-tuning. This approach successfully addressed 4 text classification tasks: clinical abbreviation disambiguation, NLI, medication attribute filling, and progress note understanding. Gu et al [85] developed AGCVT-Prompt, an LLM-based approach for analyzing health care–related social media content, with a specific focus on COVID-19 discussions. The method achieved strong performance by combining 3 key components: automatic generation of topic templates using T5 to cluster health care topics (with clustering performed via HDBSCAN [Hierarchical Density-Based Spatial Clustering of Applications with Noise] on embeddings obtained from BERT); creation of sentiment prompt templates to identify emotional content; and the use of soft prompt tokens. All components are integrated within a CoT reasoning framework.

Comparison across pretraining, fine-tuning, and prompt-tuning approaches (Tables S4-S9 in Multimedia Appendix 3) reveals a clear resource-performance trade-off. Pretraining achieved high performance across 3 applications (ie, clinical decision support, patient safety and risk assessment, and other annotations) but required extensive computational resources, which limited its adoption. Approaches ranged from massive model scales (eg, GatorTron-large) to more targeted adaptations (eg, RAD-BERT, MeDeBERTa, RuPool-BERT). Fine-tuning emerges as the dominant methodology, delivering strong performance across diverse tasks while requiring moderate computational resources compared with pretraining, particularly when PEFT techniques are leveraged. Prompt-tuning achieves competitive results by training only soft prompts, thereby requiring significantly less data and computation than full fine-tuning. In this scenario, the patterns for selecting LLMs differ substantially. While BERT and its variants dominate fine-tuning, domain-specific models such as ERNIE-Health and GatorTronGPT (and their variants) were leveraged in prompt-tuning for their preexisting health care knowledge. It is also noteworthy that task complexity clearly influenced the choice of methodology. Ethical considerations were strongly correlated with the selected approach as well. Most research papers involving fine-tuning employed on-premises deployment, whereas all pretraining and prompt-tuning studies used data that were either deidentified or processed on-premises.

#### Other LLM-Based Approaches

While reviewing the research papers considered, in addition to prompt engineering, pretraining, fine-tuning, and prompt-tuning, other approaches were also found to contribute to improving health care text classification. These LLM-based approaches include ensemble learning, data augmentation, and RAG.

Using an ensemble learning–based approach for LLM-based health care text classification involves combining the strengths of multiple models to enhance performance on complex and nuanced health care texts. These methods leverage various LLMs, such as BERT, GPT-4, or RoBERTa, to improve the robustness and accuracy of classifying unstructured health care data, including patient comments and EHRs. In some cases, the base models also incorporate conventional machine learning methods. The ensemble approach typically involves techniques such as bagging, boosting, or stacking, in which the outputs of individual models are combined—through majority voting, weighted averaging, or meta-learning—to produce final predictions. This approach helps mitigate the biases and limitations of single models, resulting in higher classification performance.

Five research studies employed ensemble learning, which leverages the strengths of multiple traditional machine learning models and LLMs. Li et al [41] used an ensemble method that combined 3 LLMs (BERT, RoBERTa, and ClinicalBERT), with the majority voting for the final classification of AD signs and symptoms in EHRs. The tasks included binary classification (ie, whether the text contains AD signs and symptoms) and multiclass classification based on 9 predefined AD signs and symptoms categories. This ensemble was trained (ie, each LLM was fine-tuned) on a combination of 3 datasets: human-annotated “gold” data, LLM-annotated “silver” data from public EHRs (using LLaMA 65 billion parameters for annotation), and synthetic “bronze” data generated by GPT-4. The methodology demonstrated that combining multiple pretrained models in an ensemble, while leveraging both LLM-annotated real clinical text and LLM-generated synthetic data for training, can effectively enhance the detection of medical conditions from clinical notes. Wu et al [84] achieved state-of-the-art performance in health care text classification using a proposed ensemble learning approach that combines 3 LLMs. The methodology focused on diabetes-related patient queries, employing ChatGLM2-6B, Qwen-7B-Chat, and MacBERT in an ensemble architecture, with each model fine-tuned using different techniques (eg, LoRA, QLoRA, fast gradient method). Additionally, ChatGPT and Claude were used for data augmentation, primarily targeting difficult-to-classify text documents. The system processed patient queries through all LLMs and employed a majority voting mechanism to determine the final classification across 6 medical categories (ie, diagnosis, treatment, common knowledge, healthy lifestyle, epidemiology, and other). Jiang et al [71] proposed ALEX-L, an unconventional ensemble learning approach for analyzing health care–related social media text, which combines BERT models with LLM-based validation. This resulted in 3 tasks: COVID-19 diagnosis, sentiment analysis, and social anxiety analysis. The methodology uses BERT variants to make initial classifications and then leverages GPT-3.5 as a verification layer. The GPT-3.5 component takes BERT’s predictions and the original health care text, combines them with task-specific instructions and examples into a prompt, and then asks GPT-3.5 to verify whether there is evidence supporting the predicted label. If GPT-3.5 determines that there is insufficient evidence (returns “false”), the prediction is corrected either through manual review or automatically converted to another class. Chaichulee et al [51] achieved its best performance in analyzing bilingual (ie, Thai and English) ADR reports using an ensemble approach that combined 6 distinct models: NB-SVM (Naïve Bayes-Support Vector Machine), ULMFiT (Universal Language Model Fine-tuning), and 4 BERT variants (mBERT [Multilingual Bidirectional Encoder Representations from Transformers], XLM-RoBERTa [Cross-lingual Language Model – RoBERTa], WangchanBERTa, and AllergyRoBERTa). This comprehensive ensemble method was applied to classify free-text clinical descriptions of drug allergies into the 36 most frequently coded symptom terms from EHRs in a Thai hospital setting. The methodology used majority voting to aggregate predictions from all models, with each model contributing its unique strengths. Yang et al [90] used GPT-4 with a ZS-CoT prompting technique, combined with ensemble querying, to identify potential drug targets from biomedical literature. The authors tested this approach on COVID-19 and Nipah virus literature, where the system analyzed paper titles, abstracts, and keywords to determine whether they contained relevant drug target information. The methodology involved crafting specific prompts that included a system message, key definitions, and carefully framed questions to guide GPT-4’s analysis. The ensemble approach sent the same query 3 times and used majority voting to determine the final decision.

Using LLMs for data augmentation in health care text classification can significantly enhance model performance, particularly when labeled data are limited. This approach leverages LLMs to generate synthetic data, which can then expand the size of the training dataset. These synthetic examples mimic real-world health care texts, adding diversity and robustness to the training process. By introducing variation in the data, LLM-generated augmentation can help reduce overfitting, a common issue when working with smaller datasets. Different augmentation strategies can be employed using LLMs, including document paraphrasing, alternative descriptions of medical conditions, or even the generation of new, plausible clinical notes or patient comments. Once the dataset is augmented, a machine learning model or LLM can be trained using a supervised learning paradigm.

Only 1 paper [63] among the eligible studies was identified as using LLMs for data augmentation in text classification. Yuan et al [63] proposed LLM-PTM to improve patient-trial matching. The methodology aims to match patients’ EHRs with clinical trials while preserving patient privacy. LLM-PTM uses a data augmentation pipeline powered by LLMs, employing ChatGPT to generate augmented versions of clinical trial eligibility criteria using desensitized patient data to maintain privacy. These versions are semantically equivalent but linguistically diverse, maintaining the exact medical meaning. The augmented data, along with patients’ EHRs, are then embedded into a shared latent space using pretrained BERT models. The embedding of patient records is enhanced with a memory network to capture the sequence of medical visits, diagnoses, and treatments, while a highway network is used to encode the eligibility criteria. Once both the patient data and clinical trial criteria are embedded, the model performs patient-trial matching. It uses a contrastive loss function to maximize the similarity between patient embeddings and inclusion criteria while minimizing similarity with exclusion criteria. This enables the model to accurately determine whether a patient meets the eligibility requirements of a clinical trial. The proposed approach improved performance while maintaining patient privacy by augmenting only the publicly available trial criteria, rather than sensitive patient data.

RAG is a recently introduced technique by Lewis et al [105] that combines LLMs with information retrieval methods. Its goal is to enhance the performance of NLP tasks, including text classification. RAG is particularly useful when the internal knowledge of an LLM is insufficient to handle a task in a specific domain. The framework is based on 2 key components: retrieval and generation. RAG works by augmenting the input to an LLM with external data sources, which provide additional context during the generation phase. It is designed to help mitigate LLM hallucinations and output inconsistencies, which are particularly important in precise domains such as health care. For text classification, RAG offers several potential advantages: (1) enhancing context to improve understanding of the input text and, consequently, classification accuracy; (2) facilitating adaptation to a specific domain by providing domain-specific information for retrieval, which is especially useful when using general-purpose LLMs; and (3) improving explainability, as the retrieved information can support a more transparent classification process. Typically, an RAG pipeline involves processing the input text, retrieving relevant information from external knowledge sources using retrieval techniques such as BM25 (Best Matching 25) [106], and then augmenting the original input with the retrieved information. The LLM then uses this augmented context to generate the classification output.

Among the eligible studies in this systematic review, 1 research paper [69] leveraged RAG for health care text classification. Ramteke and Khandelwal [69] explored using GPT-family LLMs for binary stress detection from social media posts. The authors compared the performance of GPT-4 and GPT-3.5 (in zero-shot and few-shot settings) with that of conventional machine learning methods. For the conventional machine learning methods, multiple vectorization techniques (eg, term frequency–inverse document frequency, OpenAI Embedding) were also explored. To enhance few-shot learning, RAG was employed to identify semantically similar examples from the training data. This improved the prompt by retrieving relevant information using K-nearest neighbors for efficient search. LLM-based approaches, particularly GPT-4, significantly outperformed traditional machine learning models, achieving a recall exceeding 99% for stress detection, a performance level especially valuable in clinical settings.

When comparing ensemble learning, data augmentation, and RAG approaches (Tables S10-S15 in Multimedia Appendix 3), it can be concluded that these methods serve as enhancements rather than standalone methodologies, typically combined with fine-tuning or prompt engineering to address specific limitations. Ensemble learning demonstrated the most diverse implementation strategies and consistently strong performance. LLM-based data augmentation appeared in only 1 study [63], where ChatGPT was used to generate augmented clinical trial eligibility criteria while preserving patient privacy. This highlights the underexploration of a potentially valuable approach for addressing the scarcity of labeled data in health care. Similarly, RAG implementation was limited despite its theoretical advantages for incorporating external knowledge. Its near absence in the literature represents a significant gap, particularly given RAG’s demonstrated success in other domains. Regarding application domains, ensemble learning appeared across diverse areas (ie, clinical decision support, public health and opinion analysis, patient safety and risk assessment, research/literature analysis), reflecting its broad applicability. By contrast, data augmentation and RAG were applied only to clinical decision support. Although represented in a small number of studies, these approaches demonstrate significant potential.

### Performance Evaluation

In the reviewed health care text classification literature, researchers employed various performance evaluation metrics, with accuracy-related measures being predominant. These include standard accuracy (or balanced accuracy), *F*_1_-score, recall, precision, and area under the curve score. Some studies also presented confusion matrices to provide a more detailed view of classification performance. All reviewed papers used at least one of these conventional metrics to validate their proposed approaches, with the exception of 2 [49,74], in which the former opted for positive class balance and negative class balance, and the latter relied solely on confusion matrices.

In addition to accuracy-related metrics, researchers often evaluated their approaches using computational efficiency measures such as compute time, perplexity, or FLOPS (Floating-Point Operations per Second). As shown in Table 1, compute time emerged as the most commonly reported of these metrics, appearing in 9 papers. Although some studies discussed compute time, detailed time comparisons were not provided; therefore, those studies were not included in the table. Perplexity was used less frequently, appearing in only 2 papers—1 for pretraining evaluation and another for fine-tuning assessment. FLOPS received minimal attention, with only Lehman et al [57] examining this aspect. This limited focus on FLOPS is understandable in the health care domain, as this metric primarily serves model architecture comparison and hardware optimization purposes, which often require detailed architectural information that may be unavailable for many LLMs. While some authors mentioned implementation costs, these references typically lacked explicit comparisons and were therefore excluded from the analysis presented in Table 1.

**Table 1:** Performance evaluation metrics.

Performance evaluation	Research papers
Compute time	Gu et al [85] Gretz et al [96] Yogarajan et al [55] Raja et al [88] Chen et al [92] Bețianu et al [97] Guo et al [89] Chen et al [94] Yogarajan et al [60]
Perplexity	Li et al [59] Li et al [66]
Floating-point operations per second	Lehman et al [57]

### Gaps and Limitations

#### Limitations

Despite the significant advances and insights presented in the reviewed literature, several notable gaps and limitations warrant careful consideration. This section summarizes these limitations across 4 key dimensions: data-related challenges that affect the quality and reliability of findings; model-related constraints that influence the computational approaches employed; methodological limitations that impact implementation; and ethical and privacy considerations that raise critical concerns for future research and applications in health care.

#### Data-Related Challenges

**Figure 8:**
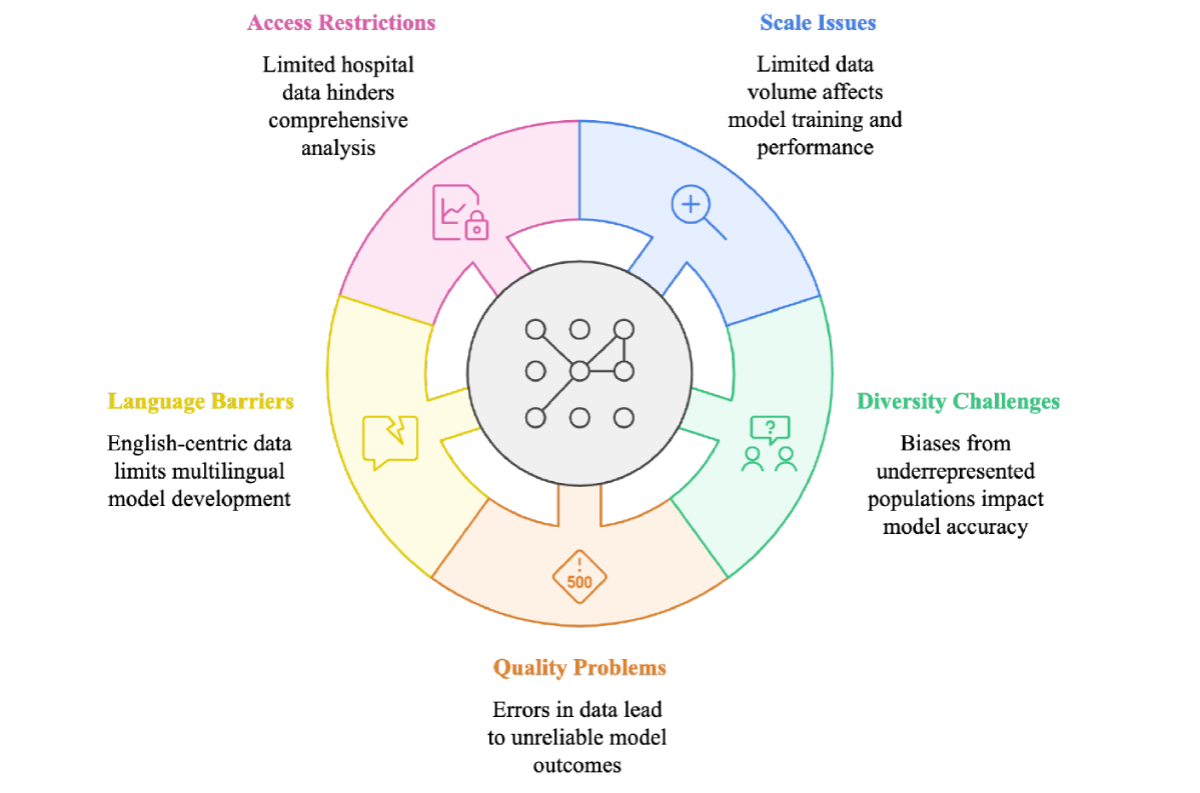
Data-related limitations.

Health care text classification using LLMs faces several significant data-related limitations, primarily concerning scale, diversity, and quality. Many studies rely on limited datasets or health care documents from single institutions, which may introduce bias and restrict the generalizability of results across different health care settings, particularly in pretraining or fine-tuning approaches. The lack of demographic diversity presents another critical challenge, as underrepresented populations may experience reduced model performance due to biases in the training data. Data quality issues further exacerbate these limitations, arising from factors such as patient deidentification errors, transcription inaccuracies, and inconsistent user-generated content. These challenges become particularly critical when dealing with minority classes that represent rare conditions, especially in fine-tuning scenarios where imbalanced datasets can significantly skew the performance of LLM-based approaches. Language constraints also pose substantial barriers, as research often focuses on English-language data, hindering the development of multilingual approaches. Additionally, researchers frequently rely on publicly available datasets (eg, MIMIC-III) due to limited access to hospital-specific data. This reliance restricts access to comprehensive patient histories and complete clinical contexts, thereby limiting the development, validation, and practical implementation of the proposed LLM-based approaches (Figure 8).

#### Model-Related Constraints

**Figure 9:**
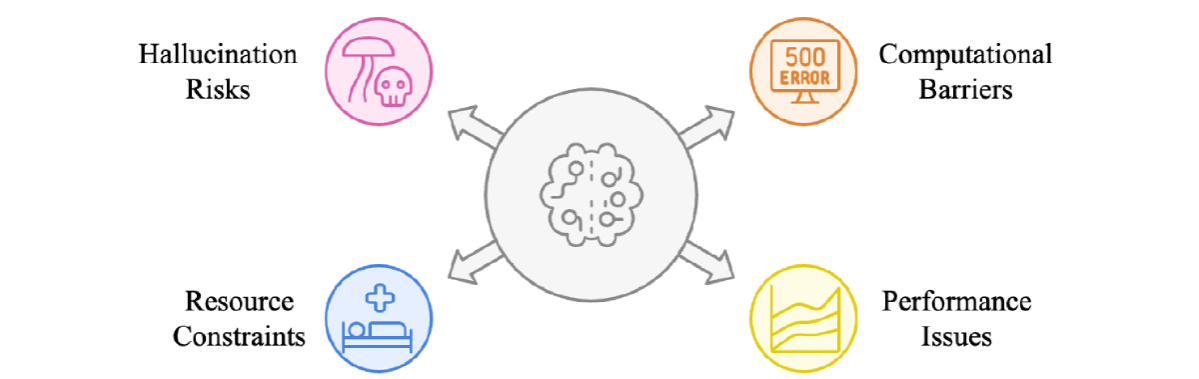
Model-related limitations.

Among the key limitations faced by LLMs, significant computational and architectural challenges can be highlighted. The complex architecture of LLMs—particularly during pretraining and fine-tuning—requires substantial computational resources. This creates considerable implementation barriers in health care settings and helps explain the prevalence of prompt engineering and BERT (or variant) fine-tuning approaches. However, even these less computationally demanding approaches face challenges. For example, processing long health care documents, especially clinical texts, is constrained by context window limitations, often necessitating chunking strategies, which can lead to slower inference speeds and potentially delay real-time applications. Performance issues are evident across several findings, including lower accuracy for rare medical conditions and challenges associated with (extreme) multilabel classification. The tendency of LLMs to hallucinate information is particularly concerning in clinical settings, where accuracy is critical. Furthermore, resource constraints within health care organizations further complicate implementation. Memory limitations often force researchers to use smaller LLM variants, which may compromise performance. Additionally, the high operational costs of advanced LLMs, such as OpenAI o1, can pose barriers to practical deployment. These technical limitations, combined with the challenges of handling complex medical jargon, further highlight the gap between current technical capabilities and the stringent requirements of health care applications (Figure 9).

#### Methodology-Related Limitations

**Figure 10:**
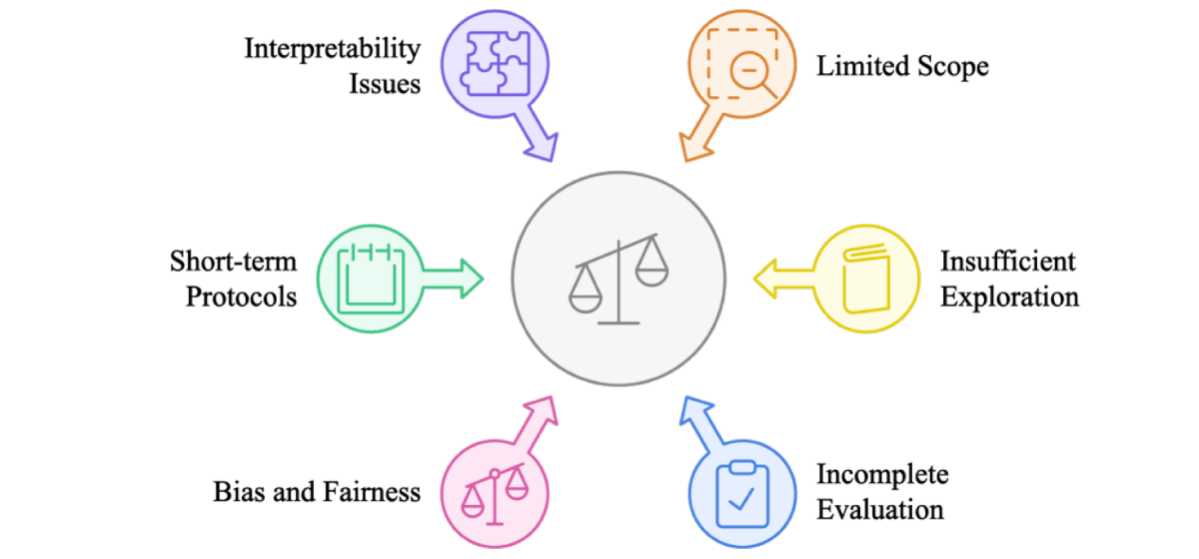
Methodology-related limitations.

Methodology-related limitations constitute another challenge for LLM-based health care text classification. These include gaps in research design, evaluation, and validation approaches. Many studies exhibit a limited scope by focusing on a single type of text classification and specific health care applications, rather than exploring a broader range of classification tasks and use cases. This narrow focus is often driven by institution-specific needs, such as addressing the requirements of individual health care facilities. Additionally, advanced techniques are frequently underexplored, and comparisons with traditional machine learning methods are limited, making it difficult to establish robust performance benchmarks. Evaluation frameworks also tend to lack comprehensiveness; limited assessment of inference latency, deployment feasibility, and operational costs represents a notable gap. As most studies prioritize accuracy over practical implementation metrics, assessing real-world feasibility in resource-constrained health care settings remains challenging. Notable gaps also include minimal attention to bias and fairness assessment. Some studies rely solely on comparisons with other LLM outputs rather than human annotations, which makes the evaluation of real-world implementation challenging. Furthermore, studies frequently lack robust, relatively long-term evaluation protocols, making it difficult to assess model stability and reliability over time. Additionally, there is often insufficient investigation of LLM interpretability, which is critical for clinical applications and represents an active area for future research. These methodological limitations ultimately affect the reliability, validity, and practical applicability of LLM-based approaches for health care text classification (Figure 10).

#### Ethical and Privacy-Related Concerns

**Figure 11:**
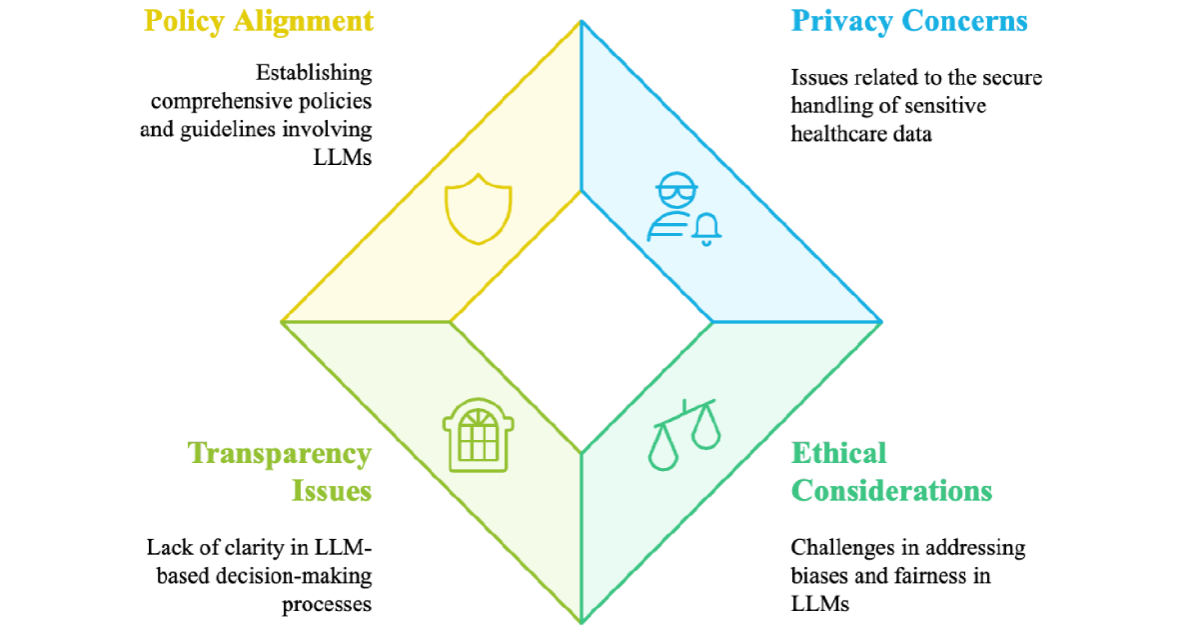
Ethical and privacy-related limitations. LLM: large language model.

Two key challenges and priority considerations in health care text classification are protecting patient privacy and ensuring ethical implementation. Privacy concerns are particularly critical when handling sensitive clinical notes using cloud-based LLMs that rely on shared computing resources and API requests. A fundamental gap lies in the limited attention given to data protection requirements and the insufficient exploration of privacy-preserving techniques, which creates potential vulnerabilities in patient data security. Furthermore, because achieving high text classification accuracy is often the primary focus of this research, ethical considerations frequently receive insufficient attention. As a result, many studies fail to address potential biases in the models and datasets used, which can lead to health care disparities. Additionally, the lack of transparency and interpretability in LLM-based decision-making processes raises ethical concerns regarding accountability and trust in clinical settings. There is also a need to address the ethical implications of automated medical decision support using LLMs, including issues of consent for data sharing and the appropriate balance between LLM-based outputs and human judgment. Moreover, given the novelty of these approaches, there is a need to establish comprehensive policies and guidelines governing the use of LLMs in health care settings (Figure 11).

### Future Research Directions

#### Toward More Robust LLMs for Health Care Text Classification

**Figure 12:**
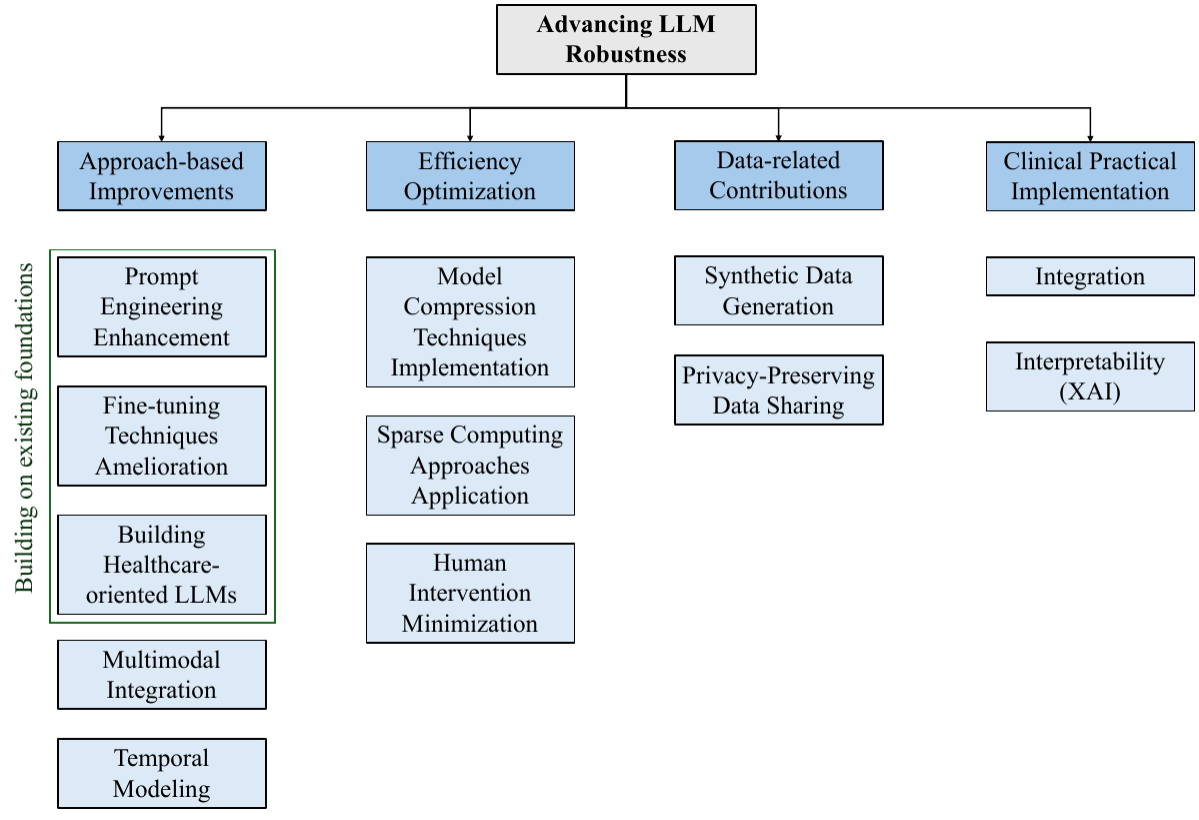
Key future research directions. LLM: large language model; XAI: explainable artificial intelligence.

The previous sections demonstrate that LLMs have shown remarkable capabilities in health care text classification; however, several gaps and limitations remain. Owing to the rapid evolution of LLM architectures and advances in computational resources, further investigation is now feasible, opening opportunities to explore areas focused on enhancing LLM robustness. These areas include approach-based improvements, efficiency optimization, data-related contributions, and clinical practical implementation, as illustrated in Figure 12. This section examines these key research directions.

#### Approach-Based Improvements

When considering future research directions, building on the existing research foundation is typically a priority. This involves focusing on approach-based improvements, including more advanced investigation of prompt engineering and fine-tuning techniques and, ideally, the development of health care–oriented LLMs from scratch (eg, GatorTron). With respect to prompt engineering enhancement, developing more sophisticated prompt strategies—such as templates that incorporate medical terminology—could be beneficial in providing richer contextual information to LLM inputs. Furthermore, smaller LLMs can be used to guide larger models hierarchically; for example, BERT can be employed to extract keywords from text documents using attention mechanisms, which can then be used to construct an enhanced prompt fed to GPT-4o. Additionally, performing text classification requires first identifying the relevant labels. In many real-world applications, such as patient comments collected through surveys, labels are initially unavailable. Topic modeling is therefore often required before proceeding with classification. An exciting research direction is to leverage LLMs for end-to-end text classification that incorporates topic modeling. Fine-tuning, which is the most commonly used LLM-based approach in the reviewed literature, is particularly useful when labeled data are limited, as is often the case. It can thus achieve high performance with minimal data requirements. However, further attention is needed to develop efficient fine-tuning techniques that fully exploit the potential of LLMs, which cannot always be adapted using standard supervised fine-tuning due to their large number of parameters. Researchers are currently addressing this challenge through PEFT [107]. Further exploration of parameter-efficient techniques suitable for resource-constrained health care environments, along with investigations into transfer learning approaches that can effectively bridge different medical domains and tasks, would be highly valuable. This remains an active research area, with PEFT techniques such as LoRA occasionally adopted in the literature when larger models are involved. Looking further ahead, developing LLMs from scratch that are trained exclusively on health care data collected from diverse sources and institutions may help reduce bias and promote fairness. These models can subsequently be adapted to different NLP tasks, such as text classification and named entity recognition. Researchers may also consider developing smaller, more focused models that excel in specific medical domains or tasks, potentially offering more practical solutions for real-world health care applications. Targeting small LMs would enable efficient fine-tuning that can be performed on-premises using local data in secure environments. Future research should also prioritize the development of robust frameworks for evaluating both accuracy and cost-efficiency.

As health care data continue to grow through the digitalization of health care processes, they increasingly span multiple modalities beyond text alone. Consequently, multimodal integration is gaining attention as a promising research direction, similar to trends observed in other industries. Although this systematic review excluded the few studies that employed multimodal data, it is important to acknowledge that health care information—particularly for clinical decision support applications—often exists in diverse forms (eg, clinical notes, medical images, lab results, vital sign readings, and structured EHR data). When integrated, these modalities can provide a more comprehensive context for LLM-based text classification. A key research challenge in multimodal integration lies in developing effective architectures capable of handling the heterogeneity of different data types while preserving their semantic relationships, thereby enabling contextually rich classification. This includes exploring fusion strategies such as early fusion, in which raw inputs are combined, and late fusion, in which each modality is processed separately and their outputs are combined at the decision level. However, expanding the range of health care data modalities may exacerbate missing data challenges, which will require specialized handling.

One research paper in the reviewed literature highlighted the study of temporal relationships in discharge summaries [44], revealing an exciting research area that focuses on capturing the dynamic and sequential nature of health care data, potentially enabling LLMs to better understand text classification applications, such as disease progression or treatment responses. The temporal dimension would add crucial context that can significantly impact the accuracy and utility of classification tasks in these specific cases. One key aspect of temporal modeling is the emphasis on enhancing LLMs’ ability to understand and process temporal expressions in clinical texts, such as temporal markers (ie, dates, times, durations) and implicit temporal relationships (ie, before, after, during) commonly present in clinical notes. Researchers could explore techniques to modify LLM architectures to better capture these temporal dependencies, such as incorporating temporal attention mechanisms that enable maintaining the chronological order of clinical events during classification tasks. Another important research avenue involves developing methods to handle longitudinal patient data. Traditional LLM approaches often treat each clinical note independently, but temporal modeling aims to maintain continuity across the patient’s medical history, which requires sophisticated architectures to track the evolution of the patient’s medical status while sustaining consistently strong classification performance. It may be worth exploring the incorporation of recurrent neural network components or temporal convolution layers within the llm architecture to capture long-term dependencies in patient histories.

### Efficiency Optimization

LLMs’ computational demands and resource requirements present significant challenges for practical implementation in health care settings, particularly during pretraining and fine-tuning. Although the literature provides proof-of-concept demonstrations of LLM applications in health care text classification, several key research directions emerge as the field evolves, especially in efficiency optimization. This section explores potential solutions, including model compression techniques, sparse computing approaches, and strategies to minimize human intervention.

Implementing model compression techniques is crucial for optimizing LLMs in health care text classification. Model compression refers to methods that reduce a model’s size and computational demands while preserving as much of its accuracy as possible. Researchers can explore specialized knowledge distillation approaches to effectively transfer health care domain knowledge from a larger model (ie, teacher) to a more compact one (ie, student) while maintaining accuracy. This area requires careful investigation of health care–specific teacher-student architectures and domain-adapted distillation techniques that preserve critical health care knowledge. Additionally, quantization can be leveraged for efficiency optimization. Quantization involves reducing the precision of an LLM’s parameters to improve memory and computational efficiency, often without a significant loss in performance. Future studies could examine mixed-precision techniques that adapt to varying health care text classification tasks, with particular attention to their impact on rare medical condition detection and diagnostic confidence scores. Posttraining quantization research can also focus on developing calibration methods specifically designed for health care text data. Furthermore, pruning strategies, which involve removing less important weights from the LLM, need to be developed with health care–aware metrics that account for the unique requirements of health care applications, particularly to maintain accuracy in detecting minority classes (eg, rare conditions).

Another approach to optimizing LLMs for health care text classification is sparse computing. Sparse computing refers to computational methods and hardware architectures designed to handle sparse data (eg, text, recommendation systems). It is particularly useful for health care text data, which often contains specialized vocabulary. Still, most words from the entire medical vocabulary are usually absent in any given text, resulting in sparse representation matrices. Additionally, in terms of distribution, health care documents generally follow Zipf’s law, where a few words or tokens appear frequently while most occur rarely. Therefore, sparse computing techniques could be particularly helpful for clinical notes, which often contain medical jargon and can be lengthy. Dynamic sparse attention mechanisms [108] may help LLMs focus on clinically relevant terms and relationships while ignoring irrelevant text, potentially improving both efficiency and accuracy. Conditional computation approaches, in which only relevant parts of the LLM are activated based on the specific health care classification task or document type, could significantly reduce computational costs.

Minimizing human intervention presents both an opportunity and a challenge that requires careful investigation. It would allow a focus on developing robust self-verification mechanisms, uncertainty quantification, and automated quality control processes, enabling LLMs to handle health care text classifications that are typically performed independently. Integrating continuous learning mechanisms would enable LLMs to adapt to evolving medical knowledge, terminology, and classification requirements. Active learning strategies could further optimize human involvement by intelligently selecting the most notable cases for expert review. This approach allows a targeted use of limited health care professional time by identifying cases in which the LLM’s confidence is low.

Additionally, converting proof-of-concept into production-level systems requires scaling the applications. For this reason, reporting deployment specifications—such as hardware, latency, and throughput—would greatly benefit the research community in efforts to build efficiently optimized LLM-based text classification approaches.

### Data-Related Contributions

As previously discussed, LLM-based health care text classification faces several data challenges, which have led to multiple research directions, including synthetic data generation and privacy-preserving data sharing. Synthetic data generation using LLMs represents a transformative approach—not only to expand research involving text in low-resource languages, but also to potentially address critical challenges in health care text classification. In health care settings, where data accessibility is often constrained by privacy regulations and ethical considerations, LLMs offer a promising solution by generating realistic textual data that can supplement existing datasets while maintaining patient confidentiality. This research direction is particularly valuable for pretraining and fine-tuning approaches. The primary advantage of LLM-based synthetic data generation lies in its ability to address data scarcity, particularly for minority classes (eg, rare medical conditions). Health care datasets often suffer from imbalanced distributions, where certain conditions or topics are underrepresented. LLMs can generate additional examples of these less frequent cases for data augmentation, helping to create more balanced datasets and improve overall classification performance. Another crucial benefit of synthetic data generation in health care is privacy preservation. Using synthetic text data in LLM-based approach development eliminates the risk of exposing patients’ sensitive information and does not require their formal consent. However, synthetic data generation presents several challenges, including ensuring medical accuracy, proper use of terminology, and addressing biases that may be inherited or even amplified from the LLM, potentially perpetuating existing disparities in health care text data. Techniques aimed at mitigating these biases warrant further investigation. Moreover, recursively training an LLM on synthetically generated data is likely to lead to model collapse [109], causing performance degradation primarily due to the model’s gradual loss of information about the distribution’s tails (ie, minority classes) and a shift toward a distribution with reduced variance. Therefore, careful consideration is needed to advance health care–oriented LLMs for text classification without being limited by access to real data, by leveraging synthetically generated health care text judiciously.

One of the previously identified gaps in the literature is the limited diversity of datasets, as texts are often collected from a single institution. Privacy-preserving data sharing between health care facilities represents a critical research direction for advancing LLM applications in health care text classification while protecting sensitive patient information. Traditional data-sharing approaches often involve the direct exposure of raw patient records, raising significant privacy concerns and legal compliance issues. Promising techniques are being developed, making privacy-preserving data sharing an active area of investigation. Federated learning [110] allows multiple health care institutions to collaboratively train LLMs on their local data without directly sharing patient records. Model parameters are aggregated centrally while the underlying training data remain distributed and private. Furthermore, differential privacy [111] techniques can be integrated into the training process to add carefully calibrated noise, preventing individual patient reidentification while preserving the population-level patterns necessary for accurate classification. The ultimate goal is to enable health care institutions to safely leverage their collective data resources through LLMs while maintaining rigorous privacy standards. This approach would accelerate improvements in LLM performance while ensuring fairness and mitigating biases across diverse health care text classification tasks and applications.

### Clinical Practical Implementation

Practical implementation of LLM-based approaches for health care text classification, particularly in clinical settings, has consistently encountered challenges. This section discusses clinical integration and interpretability (explainable artificial intelligence [XAI]) as fundamental ongoing and future research directions. Clinical integration represents a critical frontier in translating LLM capabilities into real-world health care applications for text classification tasks. One key element is understanding how health care professionals would interact with LLM systems if integrated. This includes investigating how clinicians interpret and use LLM-generated classifications and how such outputs influence clinical decision-making. Insights from these studies could inform the design of interfaces and workflows that enhance the overall process while minimizing resistance. Moreover, real-time performance is essential if LLM outputs are to assist clinical decision-making. Research in this area can explore ways to optimize inference times for LLM-based approaches while maintaining accuracy, ensuring that text classification results are available when needed for time-sensitive clinical decisions. Additionally, researchers must investigate methods to monitor system performance over time and detect potential degradation in classification accuracy. Another critical element is ensuring that LLM-based classification systems can effectively communicate with existing health care information technology infrastructure, enabling seamless information flow across platforms and departments that benefit from the LLM’s outputs. It is also important to proactively address organizational considerations, including cost-effectiveness, return on investment, and broader changes required to successfully integrate LLM-based approaches into health care delivery systems.

Practical clinical implementation of LLMs for health care text classification requires established interpretability and explainability frameworks, as these systems directly impact patient care decisions. The black-box nature of these approaches makes it challenging for health care professionals to interpret their outputs, as health care applications require transparent reasoning processes that clinicians can trust and validate. Multiple strategies should be investigated to provide more XAI for health care text classification outputs. To not only explain classifications but also generate explanations that can enhance LLM performance and trustworthiness, Wu et al [112] summarized several key strategies that researchers can use to interpret LLM text classification decisions. Attribution methods, such as gradient- and perturbation-based approaches, can help identify important input features. Component-level interpretation can be used to analyze self-attention patterns to understand how the model processes input text or to examine feed-forward networks to see how information flows through the model. Furthermore, CoT prompting, employed in some of the reviewed studies, can add explicit reasoning steps to prompts, making classification decisions more interpretable while encouraging the model to explain its reasoning in a step-by-step manner. From a practical implementation standpoint, XAI solutions must be integrated into health care workflows through interactive explanation interfaces that allow health care professionals to understand the model’s decisions at varying levels of detail. Additionally, explanations must be generated in real time to support time-sensitive clinical decisions.

### Conclusions

LLMs have demonstrated remarkable potential in health care text classification, achieving high-performance metrics, particularly those related to accuracy. Evidence shows that these models consistently outperform traditional machine learning approaches in handling complex medical text classification tasks, with particular strength in understanding context and medical terminology. Researchers have explored a range of approaches, from lightweight prompt engineering to moderately intensive prompt-tuning and fine-tuning, as well as resource-intensive methods such as pretraining, in addition to other LLM-based strategies such as ensemble learning and RAG. This diversity of approaches underscores health care text classification as an active area of LLM research. A key advantage of LLM-based approaches is their ability to enable rapid deployment without requiring extensive labeled datasets or large numbers of contributors. This is particularly valuable in health care settings, where annotated training data are often scarce and computational resources may be limited. However, several gaps and limitations must be acknowledged. For instance, the black-box nature of these models presents challenges for interpretability—a crucial factor in health care applications, where understanding decision-making processes is essential, particularly in clinical contexts. Additionally, the sensitive nature of patient data presents a persistent challenge that requires careful consideration. These represent only a subset of the identified limitations. Potential future research directions were also discussed in this systematic review, aimed at addressing these gaps. As research progresses, focusing on overcoming the current limitations of health care text classification while maintaining high performance will be crucial—not only for realizing the full potential of LLMs but also as a trade-off that researchers and health care professionals must carefully navigate.

## Appendix

Multimedia Appendix 1 PRISMA 2020 checklist.

Multimedia Appendix 2 The distribution of LLM types used for different text classification tasks and the publication type of the eligible papers included in this systematic review.

Multimedia Appendix 3 The eligible research papers' summary tables.

## Abbreviations

AD: Alzheimer disease
ADR: adverse drug reaction
API: application programming interface
BART: Bidirectional and Auto-Regressive Transformers
BC7LitCovid: BioCreative VII Literature COVID-19 Track
BERT: Bidirectional Encoder Representations from Transformers
BIOASQ: Biomedical Semantic Question Answering
BioBERT: Biomedical Bidirectional Encoder Representations from Transformers
BLURB: Biomedical Language Understanding & Reasoning Benchmark
BM25: Best Matching 25
CanMEDS: Canadian Medical Education Directions for Specialists
CHIP-CTC: Chinese Health Information Processing – Clinical Trial Classification
CLIP: Clinical Language Inference for Patient Monitoring
CoT: chain-of-thought
CT: computed tomography
DeBERTa: Decoding-enhanced Bidirectional Encoder Representations from Transformers
DRG: Diagnosis-Related Group
ED: emergency department
EHR: electronic health record
ERNIE: Enhanced Representation through Knowledge Integration – Health
FLAN: Fine-Tuned Language Net
FLOPS: Floating-Point Operations per Second
GRID-HAMD-17: GRID Hamilton Depression Rating Scale (17-item)
HDBSCAN: Hierarchical Density-Based Spatial Clustering of Applications with Noise
HIPAA: Health Insurance Portability and Accountability Act
HPV: human papillomavirus
ICD: International Classification of Diseases
LaMDA: Language Model for Dialogue Applications
LLaMA: Large Language Model Meta AI
LLM: large language model
LM: language model
LoRA: Low-Rank Adaptation
mBERT: Multilingual Bidirectional Encoder Representations from Transformers
MEDNLI: Medical Natural Language Inference
MIMIC-CXR: Medical Information Mart for Intensive Care Chest X-Ray
MS-CXR-T: Multimodal Semantic Chest X-ray—Temporal
NB-SVM: Naïve Bayes-Support Vector Machine
NLI: natural language inference
NLM: neural language model
NLP: natural language processing
PaLM: Pathways Language Model
PEFT: parameter-efficient fine-tuning
PHQ-8: 8-item Patient Health Questionnaire
PLM: pretrained language model
PRISMA: Preferred Reporting Items for Systematic Reviews and Meta-Analyses
QIC: Query Intent Classification
QLoRA: Quantized Low-Rank Adaptation
RadNLI: Radiology Natural Language Inference
RAG: Retrieval-Augmented Generation
SAE: serious adverse event
SART: Sustained Attention to Response Task
SciELO: Scientific Electronic Library Online
SDoH: Social Determinants of Health
SimCSE: Simple Contrastive Sentence Embeddings
SLM: statistical language model
TransformerXL: Transformer with Extra-Long Context
UCSF: University of California, San Francisco
ULMFiT: Universal Language Model Fine-tuning
XAI: explainable artificial intelligence
XLM-RoBERTa: Cross-lingual Language Model – RoBERTa
XLM-RoBERTa-large: Cross-lingual Language Model – RoBERTa (Large)
ZS-CoT: Zero-Shot Chain-of-Thought
