# Supplementary material for: Large Language Models for Health Care Text Classification: Systematic Review
Source: JMIR AI. 2026 Feb 11;5:e79202. doi: 10.2196/79202 (PMC12936667; doi:10.2196/79202)
Supplement: Multimedia Appendix 2 [file ai_v5i1e79202_app2.pdf]

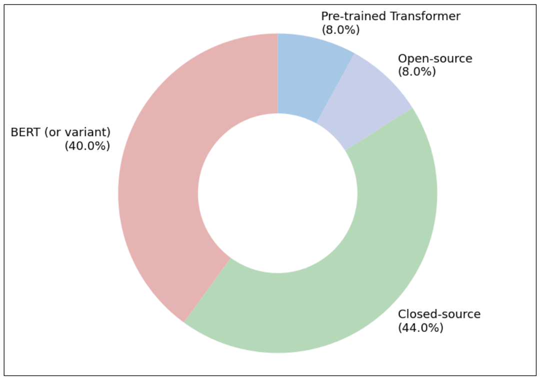

Figure S1. Types of LLMs Used for Binary

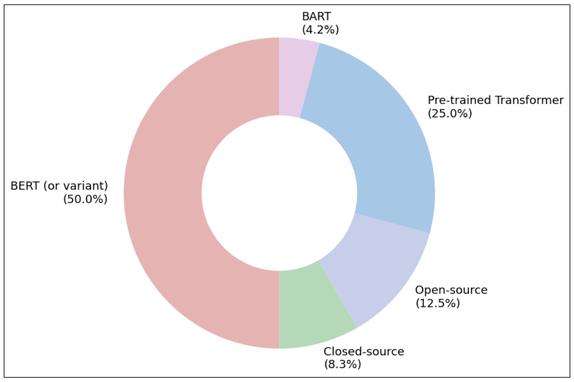

Figure S2. Types of LLMs Used for Multi-label

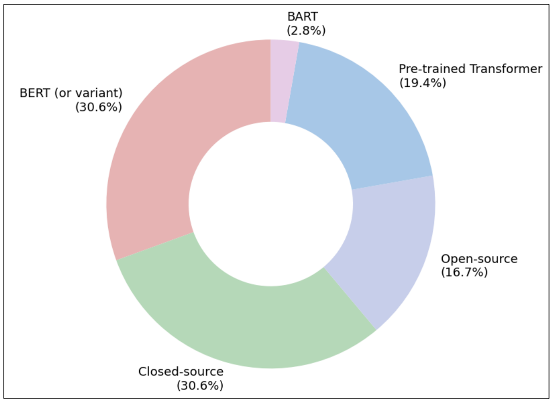

Figure S3. Types of LLMs Used for Multi-class

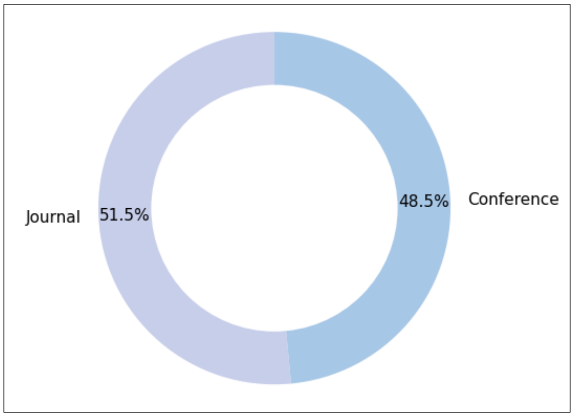

Figure S4. Types of Eligible Papers
