# Supplementary material for: Large Language Models for Health Care Text Classification: Systematic Review
Source: JMIR AI. 2026 Feb 11;5:e79202. doi: 10.2196/79202 (PMC12936667; doi:10.2196/79202)
Supplement: Multimedia Appendix 3 [file ai_v5i1e79202_app3.pdf]

Table S1. Healthcare Text Data-based Categorization of Reviewed Literature

Reference	Healthcare Text Data Type			Ethical Considerations		
	**Clinical Notes**	**Healthcare Communications**	**Research/Literature**	**On-premises Deployment**	**De-identification**	**Cloud-based Deployment**
Sushil et al. (2024)	✓				✓	✓
Lossio-Ventura et al. (2024)		✓			✓	
Shi et al. (2023)			✓			
Li et al. (2023a)	✓			✓	✓	✓
Chen et al. (2024a)			✓			
Ohse et al. (2024)	✓				✓	
BT and Chen (2024)	✓					
Aldeen et al. (2023)		✓				
Liu et al. (2023)	✓					✓
Ramteke and Khandelwal (2023)		✓				
Kim et al. (2024)		✓				
Gu et al. (2024)		✓		✓		
Raja et al. (2024)			✓			
Guo et al. (2024)			✓			
Yang et al. (2024)			✓			
Chang et al. (2024)	✓			✓		
Carneros-Prado et al. (2023)		✓				
Peng et al. (2024)	✓				✓	
Sivarajkumar and Wang (2022)	✓			✓	✓	
Wang et al. (2023)		✓	✓		✓	
Xu et al. (2024)		✓				
Williams et al. (2024)	✓				✓	
Alsentzer et al. (2023)	✓			✓		
Sarkar et al. (2023)			✓			
Yuan et al. (2023)	✓				✓	
Kementchedjhieva and Chalkidis (2023)	✓		✓		✓	
Chen et al. (2023)			✓	✓		
Yogarajan et al. (2022a)	✓				✓	
Bețianu et al. (2024)			✓	✓		
Farruque et al. (2024)		✓		✓		

Table S1. Cont.

Reference	Healthcare Text Data Type			Ethical Considerations		
	Clinical Notes	Healthcare Communications	Research/Literature	On-premises Deployment	De-identification	Cloud-based Deployment
Wu et al. (2023)		✓		✓		
Yang et al. (2022)	✓		✓	✓	✓	
McMaster et al. (2023)	✓			✓		
Guevara et al. (2024)	✓			✓	✓	
Li et al. (2023b)	✓				✓	
Li et al. (2024b)	✓				✓	
Lehman et al. (2023)	✓			✓	✓	
Schneider et al. (2021)	✓		✓		✓	
Gretz et al. (2023)			✓	✓		
Savage et al. (2023)	✓			✓	✓	
Shiju et al. (2022)		✓		✓		
Xie et al. (2024)	✓			✓		
Chen et al. (2022)			✓	✓		
Bumgardner et al. (2024)	✓			✓		
Cui et al. (2023)	✓			✓	✓	
Van Ostaeyen et al. (2023)		✓		✓		
Jiang et al. (2023)		✓				
Ge et al. (2023)		✓				
Ren et al. (2023)		✓		✓		
Wang et al. (2024)	✓				✓	
Chen et al. (2024b)			✓	✓		
Bressem et al. (2020)	✓			✓		
Tan et al. (2023)	✓				✓	
Silverman et al. (2024)	✓				✓	
Pan et al. (2020)	✓			✓		

Table S1. Cont.

Reference	Healthcare Text Data Type			Ethical Considerations		
	Clinical Notes	Healthcare Communications	Research/Literature	On-premises Deployment	De-identification	Cloud-based Deployment
Bansal et al. (2023)		✓		✓	✓	
Chaichulee et al. (2022)	✓			✓	✓	
Uslu et al. (2024)	✓			✓	✓	
Blinov et al. (2020)	✓				✓	
Yogarajan et al. (2022b)	✓				✓	
Qi et al. (2023)			✓	✓		
Ciobotaru and Dinu (2023)		✓		✓		
Luo et al. (2024)		✓	✓			
Kersting et al. (2023)		✓		✓		
Yogarajan et al. (2021)	✓			✓	✓	

Table S2. Prompt Engineering-based Reviewed Literature Categorization

Reference	Type	Language	Best LLM	Application	Performance Evaluation Accuracy				
					Accuracy	F1-score	Recall	Precision	AUC-score
Sushil et al. (2024)	Journal	English	GPT-4	Information Extraction	-	Macro: 86%	-	-	-
Lossio-Ventura et al. (2024)	Journal	English	GPT-3.5	Public Health and Opinion Analysis	NIH: 86% Stanford: 87.40%	NIH: 86.68% Stanford: 86.62%	NIH: 89.26% Stanford: 87.79%	NIH: 85.26% Stanford: 86.32%	-
Shi et al. (2023)	Conference	English	ChatGraph (w/ TF-IDF)	Research/Literature Analysis	63.63%	-	-	-	-
Chen et al. (2024a)	Journal	English	GPT-3.5-turbo-0613	Research/Literature Analysis	90.75%	-	-	-	-
Ohse et al. (2024)	Journal	English	GPT-4	Clinical Decision Support	-	73%	72%	78%	-
BT and Chen (2024)	Journal	English	AD: BARD CN: GPT-4	Clinical Decision Support	AD: 58% CN: 41%	AD: 71% CN: 61%	AD: 89% CN: 53%	AD: 60% CN: 73%	-
Aldeen et al. (2023)	Conference	English	GPT-4	Public Health and Opinion Analysis	89.9%	89.8%	-	-	-
Liu et al. (2023)	Conference	English	GPT-4	Clinical Decision Support Task 1	-	Micro: 97.9% Macro: 97.5%	-	-	-
				Clinical Decision Support Task 2	Macro Pl. eff.: 98.7% Cons.: 95.7% PNA: 96.4% PTX: 99.4% Edema: 96.8%	-	-	-	-
				Other Annotations Task 1	MS-CXR-T: 97.2% RadNLI: 93.8%	-	-	-	-
				Other Annotations Task 2	-	RadNLI: 89.2%	-	-	-
Kim et al. (2024)	Journal	English	GPT-3.5-turbo-0613	Public Health and Opinion Analysis	LF: 76.1% SF: 70.7%	-	-	-	-
Guo et al. (2024)	Journal	English	GPT-3.5/ GPT-4	Research/Literature Analysis	91%	Macro: 60%	Included: 76% Excluded: 91%	-	-

Table S2. Cont.

Reference	Type	Language	Best LLM	Application		Performance Evaluation Accuracy				
						Accuracy	F1-score	Recall	Precision	AUC-score
Raja et al. (2024)	Journal	English	BART	Research/ Literature Analysis	Article Type	91%	92%	91%	93%	91%
					Ocular Diseases	85%	85%	86%	89%	92%
					Automated Studies	92%	92%	92%	94%	95%
					DEye	63%	79%	63%	67%	60%
					DemL	99%	99%	99%	99%	98%
Chang et al. (2024)	Conference	English	Med42-70B	Clinical Decision Support		-	T (Macro): 78%<br>N (Macro): 82%<br>M (Macro): 62%	T (Macro): 77%<br>N (Macro): 81%<br>M (Macro): 62%	T (Macro): 80%<br>N (Macro): 84%<br>M (Macro): 62%	-
Carneros-Prado et al. (2023)	Conference	English	GPT-3.5	Public Health and Opinion Analysis		-	-	-	-	-
Sivarajkumar and Wang (2022)	Conference	English	HealthPrompt+ Clinical BERT	Clinical Decision Support		85%	86%	86%	86%	-
Williams et al. (2024)	Journal	English	GPT-4	Clinical Decision Support		10,000 pairs: 89%<br>500 pairs: 88%	-	-	-	-
Sarkar et al. (2023)	Conference	English	ChatGPT-3.5	Research/ Literature Analysis		-	60.60%	-	-	-
Alsentzer et al. (2023)	Journal	English	Flan-T5-XXL	Clinical Decision Support		Tone: 95.70%<br>Tissue: 91.80%<br>Trauma: 80.80%<br>Thrombin: 95.20%	Tone: 94.70%<br>Tissue: 88.70%<br>Trauma: 74%<br>Thrombin: 50%	Tone: 93.10%<br>Tissue: 82.70%<br>Trauma: 67.10%<br>Thrombin: 38.50%	-	-

Table S3. Text Classification and LLM Type Categorization: The Case of Prompt Engineering

Reference	Text Classification Type			Large Language Model Type				
	Multi-class	Multi-label	Binary	BERT (or variant)	Closed-source	Open-source	Pre-trained Transformer	BART
Sushil et al. (2024)	✓	✓			✓			
Lossio-Ventura et al. (2024)	✓				✓			
Shi et al. (2023)	✓				✓			
Chen et al. (2024a)	✓				✓			
Ohse et al. (2024)			✓		✓			
BT and Chen (2024)	✓				✓			
Aldeen et al. (2023)			✓		✓			
Liu et al. (2023)	✓		✓		✓			
Kim et al. (2024)	✓				✓			
Raja et al. (2024)	✓	✓						✓
Guo et al. (2024)			✓		✓			
Chang et al. (2024)	✓					✓		
Carneros-Prado et al. (2023)	✓				✓			
Sivarajkumar and Wang (2022)	✓			✓				
Williams et al. (2024)			✓		✓			
Alsentzer et al. (2023)	✓						✓	
Sarkar et al. (2023)		✓			✓			

Table S4. Fine-tuning-based Reviewed Literature Categorization

Reference	Type	Language	Best LLM	Application	Performance Evaluation Accuracy				
					Accuracy	F1-score	Recall	Precision	AUC-score
Ohse et al. (2024)	Journal	English	GPT-3.5	Clinical Decision Support	-	82%	84%	85%	-
Raja et al. (2024)	Journal	English	BioBERT	Research/ Literature Analysis	-	Micro: 67%	-	-	70%
Xu et al. (2024)	Conference	English	Mental-Alpaca	Clinical Decision Support	Task 1 (Dreaddit): 81.60%<br>Task 2 (DepSeverity): 77.50%<br>Task 4: 72.40%<br>Task 2 (Red-Sam): 60.40%<br>Task 1 (SAD): 81.90%	-	-	-	-
			Mental-FLAN-T5		Task 3: 75.56%<br>Task 5: 86.80%<br>Task 6: 48.10%<br>Task 2 (Twt-60Users): 73.60%	-	-	-	-
Guevara et al. (2024)	Journal	English	Flan-T5 XXL + Synth. data	Quality and Equity	-	RT (Any SDoH): 70%	-	-	-
					-	Imm. (Any SDoH): 71%	-	-	-
			Flan-T5 XL + Synth. data		-	Imm. (Adverse SDoH): 66%	-	-	-
			Flan-T5 XXL		-	MIMIC-III (Any SDoH): 57%	-	-	-
			Flan-T5 XL		-	RT (Adverse SDoH): 69%	-	-	-
					-	MIMIC-III (Adverse SDoH): 53%	-	-	-
Li et al. (2024b)	Conference	English	LlamaCare	Patient Safety and Risk Assessment	-	-	-	-	MOR: 77.62%
					-	-	-	-	LOS: 68.76%
				Clinical Decision Support	-	-	-	-	DIAG: 79.16%
					-	-	-	-	PROC: 90.76%

Table S4. Cont.

Reference	Type	Language	Best LLM	Application	Performance Evaluation Accuracy				
					Accuracy	F1-score	Recall	Precision	AUC-score
Lehman et al. (2023)	Conference	English	BioClinRoBERTa	Information Extraction	-	Macro: 70.70% Micro: 80.50%	-	-	-
				Other Annotations	90%	-	-	-	-
Schneider et al. (2021)	Conference	Portuguese	GPT2-Bio-Pt	Patient Safety and Risk Assessment	-	Weighted: 90.09%	-	-	-
Gretz et al. (2023)	Conference	English	Flan-T5-XXL	Research/Literature Analysis	-	Macro: 60.69%	-	-	-
Savage et al. (2023)	Journal	English	BioMed-RoBERTa	Clinical Decision Support	-	-	67%	-	PR: 82% ROC: 89%
Shiju et al. (2022)	Conference	English	Bio_ClinicalBERT	Public Health and Opinion Analysis	87%	87%	-	-	-
Xie et al. (2024)	Journal	English	Clinical_BERT	Clinical Decision Support Quality and Equity	-	-	-	-	-
Chen et al. (2022)	Journal	English	LitMC-BERT	Research/Literature Analysis	LitCovid: 80.22% HoC: 68.54%	LitCovid: 93.14% HoC: 90.36%	LitCovid: 92.12% HoC: 90.38%	LitCovid: 94.18% HoC:90.35%	-
Bumgardner et al. (2024)	Conference	English	Path-LLaMA 13B	Clinical Decision Support	74.80%	77.50%	77.70%	77.90%	81.60%
Cui et al. (2023)	Conference	English	BERT	Clinical Decision Support	77.56%	92.45%	-	-	-
Van Ostaeyen et al. (2023)	Journal	Dutch	RobBERT	Quality and Equity	-	Quality: 76% CanMEDS: 72%	-	-	-
Ge et al. (2023)	Conference	Chinese	Baichuan2-13B	Patient Query Analysis	92.10%	-	-	-	-
Ren et al. (2023)	Conference	English	BERTweet	Patient Query Analysis	78%	Macro: 75%			
Wang et al. (2024)	Journal	English	DRG-LLaMA-7B	Clinical Decision Support	ACC@1: 52.00% ACC@5: 84.80% ACC@10: 91.20%	Macro: 32.70%	-	-	Macro: 98.60% Micro:99.40%

Table S4. Cont.

Table 4. Cont.

**Reference**	**Type**	**Language**	**Best LLM**	**Application**	**Performance Evaluation Accuracy**				
					**Accuracy**	**F1-score**	**Recall**	**Precision**	**AUC-score**
Chen et al. (2024b)	Journal	English	BioBERT	Research/Literature Analysis	-	Discussion: 80%<br>Unstructured: 82.10%<br>Structured: 90/20%	-	-	-
Tan et al. (2023)	Journal	English	GatorTron	Clinical Decision Support	86.09%	-	-	-	-
Silverman et al. (2024)	Journal	English	H-UCSF-BERT	Patient Safety and Risk Assessment	Task 1: 88%	Task 1: 62%	Task 1: 38%	Task 1: 27%	-
			H-UCSF-BERT + only nearby SAEs		Task 2: 92%<br>Task 3: 91%	Task 2: 68%<br>Task 3: 61%	Task 2: 36%<br>Task 3: 44%	Task 2: 45%<br>Task 3: 19%	-
Kementchedjhieva and Chalkidis (2023)	Conference	English	T5Enc	Clinical Decision Support	-	Micro: 60.50%<br>Macro: 31.10%	-	-	-
				Research/Literature Analysis	-	Micro: 75.10%<br>Macro: 66%	-	-	-
Yogarajan et al. (2022a)	Conference	English	TransformerXL	Clinical Decision Support	-	Micro: 72.30%<br>Macro: 67.70%	-	-	-
Bețianu et al. (2024)	Conference	English	DALLMi-BERT	Research/Literature Analysis	-	-	-	mAP: 58.20%	-
Farruque et al. (2024)	Journal	English	Mental-BERT	Clinical Decision Support	-	DSD (Macro): 45%<br>DSD (Weighted): 56%	DSD (Macro): 51%<br>DSD (Weighted): 68%	DSD (Macro): 49%<br>DSD (Weighted): 51%	-
					-	DPD: 89%	DPD: 97%	DPD: 83%	-
Pan et al. (2020)	Conference	Chinese	FAMLC-BERT	Clinical Decision Support	-	100%	65.39%	79.07%	99.90%
Bansal et al. (2023)	Conference	English	DeBERTa Large	Public Health and Opinion Analysis	-	Macro: 67%	-	-	-
Uslu et al. (2024)	Journal	English	CXR-BERT-GENERAL	Clinical Decision Support	-	Macro: 71.61%<br>Micro: 80.49%<br>Weighted: 80.14%	Macro: 69.91%<br>Micro: 80.73%<br>Weighted: 80.73%	Macro: 75.44%<br>Micro: 80.27%<br>Weighted: 80.22%	-
Qi et al. (2023)	Conference	English	SaFER-BERT	Research/Literature Analysis	Task 1: 80%<br>Task 2: 94%	-	-	-	-

Table S4. Cont.

Reference	Type	Language	Best LLM	Application	Performance Evaluation Accuracy				
					**Accuracy**	**F1-score**	**Recall**	**Precision**	**AUC-score**
Ciobotaru and Dinu (2023)	Conference	Romanian	Romanian BERT	Public Health and Opinion Analysis	84%	Macro: 84%	Macro: 84%	Macro: 85%	-
Chen et al. (2023)	Journal	English	BioLinkBERT-Large	Research/ Literature Analysis	-	Micro: 84.90%	-	-	-
Yogarajan et al. (2022b)	Conference	English	TransformerXL	Patient Safety and Risk Assessment	-	-	MIMIC-III (Macro): 51% MIMIC-III (Micro): 65% eICU (Macro): 40% eICU (Micro): 63%	-	-
Luo et al. (2024)	Journal	English & Chinese	Taiyi (Fine-tuned Qwen-7B-base)	Research/Literature Analysis	-	BC7LitCoivd (Micro): 84% HoC (Micro): 80%	-	-	-
				Patient Query Analysis	-	KUAKE_QIC (Micro): 77.4%	-	-	-
Kersting et al. (2023)	Journal	German	XLM-RoBERTa-large	Public Health and Opinion Analysis	A: 88% B: 86% C: 88% D: 89%	A: 60% B: 45% C: 57% D: 68%	A: 57% B: 44% C: 55% D: 71%	A: 72% B: 50% C: 60% D: 67%	-
Yogarajan et al. (2021)	Conference	English	PubMedBERT	Clinical Decision Support	-	MIMIC-III (Micro): 65% MIMIC-III (Macro): 41%	-	-	-
			BioMed-RoBERTa-base		-	eICU (Micro): 60% eICU (Macro): 32%	-	-	-

Table S5. Pre-training-based Reviewed Literature Categorization

Reference	Type	Language	Best LLM	Application	Performance Evaluation Accuracy				
					Accuracy	F1-score	Recall	Precision	AUC-score
Yang et al. (2022)	Journal	English	GatorTron-large	Other Annotations	90.20%	-	-	-	-
McMaster et al. (2023)	Journal	English	MeDeBERTa	Patient Safety and Risk Assessment	-	61.10%	-	-	PR: 89.70% ROC: 95.59%
Li et al. (2023b)	Journal	English	Clinical-Longformer	Clinical Decision Support	OpenI: 97.70%	MIMIC-AKI:48.40%	-	-	MIMIC -AKI: 76.20%
				Other Annotations	MedNLI: 84.20%	-	-	-	-
Bressem et al. (2020)	Journal	German	RAD-BERT	Clinical Decision Support	89%	90%	-	-	PR:93% ROC: 98%
Blinov et al. (2020)	Conference	Russian	RuPool-BERT	Clinical Decision Support	-	Macro: 29.83% Weighted: 47.13%	-	-	-

Table S6. Prompt-tuning-based Reviewed Literature Categorization

Reference	Type	Language	Best LLM	Application	Performance Evaluation Accuracy				
					Accuracy	F1-score	Recall	Precision	AUC-score
Wang et al. (2023)	Journal	Chinese	ERNIE-Health	Patient Query Analysis	86.60%	-	-	-	-
				Research/ Literature Analysis	86.10%	-	-	-	-
Peng et al. (2024)	Journal	English	GatorTronGPT-5B	Information Extraction	Clinical Abbreviation Disambiguation	-	98.42%	98.32%	98.54%
			GatorTronGPT-20B		Natural Language Inference	89.46%	-	-	-
			GatorTronGPT		Medication Attribute Filling	-	Event: 93.79% Context: 91.26%	-	-
			GatorTronGPT-20B		Progress Note Understanding	-	79.54%	-	-
Gu et al. (2024)	Journal	Chinese	T5-AGCVT-Prompt	Public Health and Opinion Analysis	79%	75%	-	-	-

Table S7. Text Classification and LLM Type Categorization: The Case of Fine-tuning

Reference	Text Classification Type			Large Language Model Type				
	Multi-class	Multi-label	Binary	BERT (or variant)	Closed-source	Open-source	Pre-trained Transformer	BART
Ohse et al. (2024)			✓		✓			
Raja et al. (2024)		✓		✓				
Xu et al. (2024)	✓		✓			✓	✓	
Guevara et al. (2024)		✓					✓	
Li et al. (2024b)	✓	✓	✓			✓		
Lehman et al. (2023)	✓	✓		✓				
Schneider et al. (2021)			✓		✓			
Gretz et al. (2023)	✓						✓	
Savage et al. (2023)			✓	✓				
Shiju et al. (2022)			✓	✓				
Xie et al. (2024)			✓	✓				
Bansal et al. (2023)		✓		✓				
Uslu et al. (2024)		✓		✓				
Yogarajan et al. (2022b)		✓					✓	
Qi et al. (2023)			✓	✓				
Ciobotaru and Dinu (2023)	✓			✓				
Luo et al. (2024)		✓				✓		
Kersting et al. (2023)	✓			✓				
Yogarajan et al. (2021)		✓		✓				
Chen et al. (2022)		✓		✓				

Table S7. Cont.

Reference	Text Classification Type			Large Language Model Type				
	**Multi-class**	**Multi-label**	**Binary**	**BERT (or variant)**	**Closed-source**	**Open-source**	**Pre-trained Transformer**	**BART**
Bumgardner et al. (2024)		✓				✓		
Cui et al. (2023)			✓	✓				
Van Ostaeyen et al. (2023)	✓	✓		✓				
Ge et al. (2023)	✓					✓		
Ren et al. (2023)	✓			✓				
Wang et al. (2024)	✓					✓		
Chen et al. (2024b)	✓			✓				
Tan et al. (2023)	✓				✓			
Silverman et al. (2024)			✓	✓				
Kementchedjhieva and Chalkidis (2023)		✓					✓	
Chen et al. (2023)		✓		✓				
Yogarajan et al. (2022a)		✓					✓	
Bețianu et al. (2024)		✓		✓				
Farruque et al. (2024)	✓	✓	✓	✓				
Pan et al. (2020)		✓		✓				

Table S8. Text Classification and LLM Type Categorization: The Case of Pre-training

Reference	Text Classification Type			Large Language Model Type				
	Multi-class	Multi-label	Binary	BERT (or variant)	Closed-source	Open-source	Pre-trained Transformer	BART
Yang et al. (2022)	✓						✓	
McMaster et al. (2023)			✓	✓				
Li et al. (2023b)	✓	✓	✓				✓	
Bressem et al. (2020)		✓		✓				
Blinov et al. (2020)	✓			✓				

Table S9. Text Classification and LLM Type Categorization: The Case of Prompt-tuning

Reference	Text Classification Type			Large Language Model Type				
	Multi-class	Multi-label	Binary	BERT (or variant)	Closed-source	Open-source	Pre-trained Transformer	BART
Wang et al. (2023)	✓						✓	
Peng et al. (2024)	✓				✓			
Gu et al. (2024)	✓						✓	

Table S10. Ensemble Learning-based Reviewed Literature Categorization

Reference	Type	Language	Best LLM	Application	Performance Evaluation Accuracy				
					Accuracy	F1-score	Recall	Precision	AUC-score
Li et al. (2023a)	Conference	English	BERT-based Ensemble	Clinical Decision Support	Binary: 94%<br>MC: 72%	Binary: 80%<br>MC: -	Binary: 74%<br>MC: -	Binary: 86%<br>MC: -	-
Wu et al. (2023)	Conference	Chinese	MacBERT, ChatGLM2-6B, and Qwen-7B-Chat Ensemble	Patient Query Analysis	92%	-	-	-	-
Jiang et al. (2023)	Conference	English	ALEX-L	Clinical Decision Support	Task 1: 96.71%<br>Task 4: 89.84%	Task 1: 94.97%<br>Task 4: 88.17%	-<br>-	-<br>-	-<br>-
				Public Health and Opinion Analysis	77.84%	89.13%	-	-	-
Chaichulee et al. (2022)	Journal	Thai & English	NB-SVM, ULMFiT, and BERT-based Models Ensemble	Patient Safety and Risk Assessment	98.37%	98.88%	98.72%	99.31%	-
Yang et al. (2024)	Journal	English	GPT-4	Research/Literature Analysis	SARS-CoV-2:92.87%<br>Nipah:87.40%	SARS-CoV-2: 88.43%<br>Nipah: 73.90%	SARS-CoV-2: 83.38%<br>Nipah: 74.72%	-	-

Table S11. Data Augmentation-based Reviewed Literature Categorization

Reference	Type	Language	Best LLM	Application	Performance Evaluation Accuracy				
					Accuracy	F1-score	Recall	Precision	AUC-score
Yuan et al. (2023)	Conference	English	ChatGPT	Clinical Decision Support	-	Criteria: 91%<br>Trial: 81.50%	Criteria: 86.20%<br>Trial: 83%	Criteria: 96.40%<br>Trial: 80.10%	-

Table S12. Retrieval Augmented Generation-based Reviewed Literature Categorization

Reference	Type	Language	Best LLM	Application	Performance Evaluation Accuracy				
					Accuracy	F1-score	Recall	Precision	AUC-score
Ramteke and Khandelwal (2023)	Conference	English	GPT-4	Clinical Decision Support	-	-	>99%	-	-

Table S13. Text Classification and LLM Type Categorization: The Case of Ensemble Learning

Reference	Text Classification Type			Large Language Model Type				
	Multi-class	Multi-label	Binary	BERT (or variant)	Closed-source	Open-source	Pre-trained Transformer	BART
Li et al. (2023a)	✓		✓	✓				
Wu et al. (2023)	✓			✓		✓		
Jiang et al. (2023)	✓		✓	✓	✓			
Chaichulee et al. (2022)		✓		✓				
Yang et al. (2024)			✓		✓			

Table S14. Text Classification and LLM Type Categorization: The Case of Data Augmentation

Reference	Text Classification Type			Large Language Model Type				
	Multi-class	Multi-label	Binary	BERT (or variant)	Closed-source	Open-source	Pre-trained Transformer	BART
Yuan et al. (2023)	✓				✓			

Table S15. Text Classification and LLM Type Categorization: The Case of Retrieval Augmented Generation

Reference	Text Classification Type			Large Language Model Type				
	Multi-class	Multi-label	Binary	BERT (or variant)	Closed-source	Open-source	Pre-trained Transformer	BART
Ramteke and Khandelwal (2023)			✓		✓			
